# Substrates of the MAPK Slt2: Shaping Yeast Cell Integrity

**DOI:** 10.3390/jof8040368

**Published:** 2022-04-04

**Authors:** Gema González-Rubio, Lucía Sastre-Vergara, María Molina, Humberto Martín, Teresa Fernández-Acero

**Affiliations:** Departamento de Microbiología y Parasitología, Facultad de Farmacia, Universidad Complutense de Madrid, and Instituto Ramón y Cajal de Investigaciones Sanitarias (IRYCIS), 28040 Madrid, Spain; gemagonzalezrubio@ucm.es (G.G.-R.); lusastre@ucm.es (L.S.-V.); molmifa@farm.ucm.es (M.M.)

**Keywords:** yeast, phosphorylation, cell wall integrity pathway, MAPK substrate, Slt2, kinase assay

## Abstract

The cell wall integrity (CWI) MAPK pathway of budding yeast *Saccharomyces cerevisiae* is specialized in responding to cell wall damage, but ongoing research shows that it participates in many other stressful conditions, suggesting that it has functional diversity. The output of this pathway is mainly driven by the activity of the MAPK Slt2, which regulates important processes for yeast physiology such as fine-tuning of signaling through the CWI and other pathways, transcriptional activation in response to cell wall damage, cell cycle, or determination of the fate of some organelles. To this end, Slt2 precisely phosphorylates protein substrates, modulating their activity, stability, protein interaction, and subcellular localization. Here, after recapitulating the methods that have been employed in the discovery of proteins phosphorylated by Slt2, we review the bona fide substrates of this MAPK and the growing set of candidates still to be confirmed. In the context of the complexity of MAPK signaling regulation, we discuss how Slt2 determines yeast cell integrity through phosphorylation of these substrates. Increasing data from large-scale analyses and the available methodological approaches pave the road to early identification of new Slt2 substrates and functions.

## 1. Cell Wall Integrity Pathway: An Introductory View

**Figure 1:**
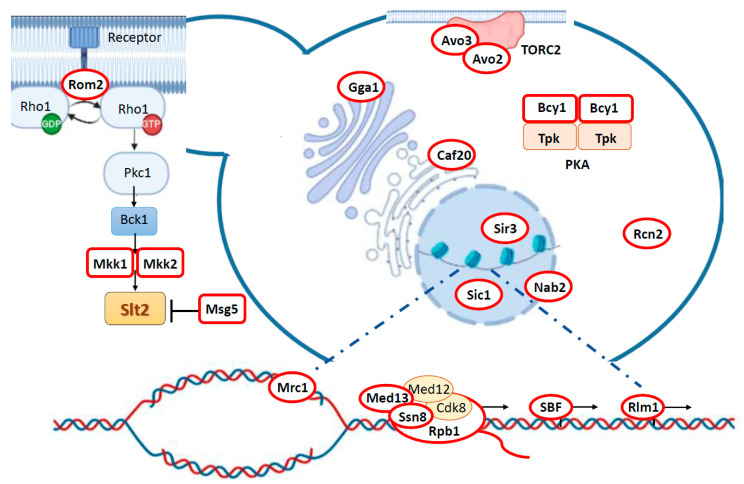
Schematic representation of CWI pathway and subcellular localization of Slt2 substrates within yeast cell. Substrates are depicted in white and encircled by a red line. The CWI pathway and most Slt2 substrates implicated in gene expression regulation can be found at the emergent bud and in an enlarged view at the bottom of the image, respectively.

All eukaryotes use signal transduction pathways mediated by mitogen-activated protein kinases (MAPKs) to adequately respond and adapt to distinct stresses and environmental changes. Upon stimulation, these pathways engage a three-tiered hierarchical phosphorylation cascade, involving the sequential activation of a MAP kinase kinase kinase (MAPKKK), a MAP kinase kinase (MAPKK), and finally a MAP kinase (MAPK). Studies over the last three decades performed by our group and others have revealed many details about the architecture, regulation, stimulation, and functionality of the cell wall integrity (CWI) MAPK pathway of budding yeast *Saccharomyces cerevisiae*. The structure of this pathway displays the typical arrangement of signaling components operating in MAPK pathways, in which a set of membrane-spanning mechanosensors (Wsc1, Wsc2, Wsc3, Mid2, and Mtl1) detect stimuli and relay signals to a GTPase (Rho1), mainly through the GDP/GTP exchange factor (GEF) Rom2 (Figure 1). One of the main effectors of Rho1 is the protein kinase Pkc1, which triggers a phosphorylation cascade composed of the MAPKKK Bck1; two redundant MAPKKs, Mkk1 and Mkk2; and the downstream MAPK Slt2 (also known as Mpk1) [1,2,3].

The concerted action of specific regulatory components and the precise molecular mechanisms of activation and inactivation ensure proper spatiotemporal control of the pathway, providing the right timing, tuning, and teaming in the signaling process. These additional components include positive regulators, such as the GEFs for Rho1, Rom1, and Tus1 [4]; and mammalian 3-phosphoinositide-dependent protein kinase 1 (PDK1) homologue kinases Pkh1 and Pkh2, which act on the protein kinase Pkc1 [5,6,7]; as well as some negative regulatory elements such as Rho1 GTPase-activating proteins (GAPs) Sac7, Bem2, and Lrg1 [2,5] and down-modulatory phosphatases. The latter consists of the serin/threonine phosphatase Ptc1, tyrosine phosphatases Ptp2 and Ptp3, and dual-specificity phosphatases (DSPs) Msg5 and Sdp1. Phosphatases ultimately modulate the phosphorylation level and thereby the activation status of Slt2, either directly or through the downregulation of upstream kinases, as occurs with Ptc1 [2,8,9,10].

Regarding the regulatory mechanisms, post-translational modifications such as phosphorylation of signaling components are fundamental for their governance [11]. Moreover, there are additional regulatory layers operating on the pathway. Among them, cell membrane surface metabolism deeply influences the functionality of the pathway; complex sphingolipids and phosphoinositol phosphates (PIPs) are essential for signaling, since interactions with these lipid species ensure the correct membrane localization of some CWI pathway components [5]. For example, Rom2 interacts through its PH domain with PI(4,5)P_2_ [12]. The chaperoning activity of Hsp90 can also be considered as an example of a fundamental regulatory level for signaling because it is essential for dually phosphorylated Slt2 to activate its downstream target Rlm1 [13].

As studies progressed, the range of stimuli found to engage the CWI pathway expanded beyond the initially identified heat shock and cell wall-specific stresses to also include plasma membrane, oxidative, and genotoxic stresses, stationary phase, protein unfolding, and low or high pH, among other conditions [2,14,15]. This means that a great variety of physical changes and chemical compounds that induce those stresses trigger the activation of the CWI pathway [15], resulting in the stimulation of Slt2-specific MAPKKs Mkk1 and Mkk2, which phosphorylate threonine (T190) and tyrosine (Y192) residues within the T-E-Y motif of the activation loop of Slt2 [16]. An alternative mode of activation has been described for Slt2 driven by genotoxic stress, which does not involve the stimulation of these MAPKKs. Instead, DNA damage activates Slt2 through the induction of proteasomal degradation of the Msg5 phosphatase [17]. Since it is widely accepted that full activation of Slt2 occurs in parallel with its dual phosphorylation, stimulation of the pathway is commonly and easily monitored using commercial antibodies that detect the dually phosphorylated form of Slt2 [16]. However, some of these antibodies are also able to detect the monophosphorylated species at either T190 or T192. The fact that some Slt2 mutants with low catalytic activity have recently been shown to display Y192 hyperphosphorylation highlights the convenience of discerning among dual and each monophosphorylated form [18].

Regarding the functional outputs, Slt2 is known to be involved in various cellular functions, including the control of cell wall biogenesis, actin cytoskeleton dynamics [2], osmolyte and metalloid transport [19], iron homeostasis [20], cell cycle progression [21], control of sphingolipid synthesis [5], mitophagy and pexophagy [22], and endoplasmic reticulum and mitochondrial inheritance [23,24]. As detailed below, Slt2 is also implicated in the regulation of its own CWI pathway by mediating feedback mechanisms [11,25,26]. Interestingly, the introduction of synthetic genetic feedback circuits that alter this autocontrol upon stimulating conditions could lead to lethality due to detrimental hyperactivation of the pathway [27]. Slt2 also modulates other signaling routes, such as the TORC2 and PKA pathways [28,29]. Most of these functions require the catalytic activity of Slt2 on its target proteins, although non-catalytic mechanisms have also been described for Slt2 that promote the induction of a subset of cell wall stress-activated genes through the SBF transcription factor [30,31].

By taking advantage of the versatility and reversibility of phosphorylation, MAPKs can modify the activity, stability, interacting properties, or subcellular localization of their substrates. MAPK substrates are therefore essential links between stimulus-triggered signal transmission and the functional outputs elicited by MAPK pathways. MAPKs are considered serine/threonine protein kinases with a requirement for proline at the +1 position of the phosphorylation residue. The phosphorylation motif recognized by mammalian MAPKs ERK1/2 can be generalized as P-X-S/T-P [32], although proline at −2 seems to be dispensable for phosphorylation and proline at +1 may be absent in particular cases [33]. Interestingly, a peptide library screening approach suggested that in addition to proline at the +1 position, and in contrast to the other yeast MAPKs, Slt2 was especially selective at the −3 position, in which the preferred residue was arginine [34]. Although the cyclin-dependent kinases (CDKs) are also proline-directed serine/threonine kinases, their consensus phosphorylation signature is S/T-P-X-K [35].

In addition to the presence of an S/T-P phosphorylation motif in the primary sequence of the substrate, which is very common in all proteins, the existence of interaction motifs between MAPK and its targets also contributes to increasing the specificity and efficiency of the phosphorylation process. Substrate binding is commonly mediated by short MAPK docking sites present in different MAPK partners. D-motifs are the most used docking sites [36], which are frequently located N-terminally from the targeted phosphorylation site [37]. The consensus sequence of D-motifs is ψ_1–3_X_3–7_ϕXϕ (where ψ is a positively charged residue, X is any residue, and ϕ is a hydrophobic residue) [37,38]. D-motifs interact with the negatively charged common docking (CD) domain and hydrophobic docking grooves at MAPKs [39,40]. Another binding motif to anchor MAPKs is the F-motif or DEF motif (docking site for ERK, FXF) [41]. While Slt2 displays a CD domain, it lacks an FXF-binding site [42].

The wide variety of stimuli and the numerous functions performed by the CWI pathway predict a high number of phosphorylation targets for Slt2. However, the complete repertoire of cellular substrates of this MAPK remains far from being elaborated. This may be why there are several reviews covering multiple aspects of this key signaling pathway, but an analysis focused on Slt2 substrates has not yet been accomplished. In this review, we aim to compile the bona fide Slt2 substrates found to date and attempt to both understand the kinase–substrate relationship and provide links between Slt2 activity and cellular functions of the CWI pathway.

## 2. Substrate Fishing: Methods Used for Searching Slt2 Targets

The order of action of genes in a regulatory hierarchy such as that governing MAPK signaling pathways can be determined by an epistasis analysis. Loss-of-function mutations in upstream components can be suppressed by either gain-of-function mutations or overexpression of downstream elements of the pathway. Conversely, loss-of-function mutations in downstream components can ameliorate defects provoked by upstream hyperactive alleles. Based on this concept, the transcription factor Rlm1 (resistance to lethality of *MKK1^P386^* overexpression) was the first Slt2 target identified as a mutant suppressor of the toxicity caused by Slt2 hyperactivation triggered by overexpression of a constitutively active mutant of *MKK1* [43]. This study positioned Rlm1 downstream of Slt2, but the confirmation of direct phosphorylation required an additional biochemical analysis [44].

Over the years, several methods for identifying MAPK substrates based on phosphorylation assays have been used in yeast studies, ranging from traditional radioactive in vitro kinase assays to a large-scale in vivo phosphoproteomic analysis. Kinase enzymes catalyze the transfer of a phosphate group from a donor ATP molecule to a substrate. Therefore, in vitro kinase assays provide a direct measurement of catalytic activity by detecting and quantifying the formation of the phosphorylated product. The possibility of using purified proteins in these assays allows the addition of large amounts of both kinase and substrate, increasing the sensitivity and avoiding spurious reactions that may occur due to the eventual presence of contaminant kinases. Typically, activated polyhistidine- or glutathione S-transferase (GST)-tagged MAPKs are purified by immunoprecipitation or affinity chromatography from yeast cells. The MAPK can be obtained in its active state by either treating the cells with the adequate stimulus or overexpressing hyperactive MAPK-activating proteins. Tagged substrates are usually expressed and purified from either *Escherichia coli* or yeast cells. Protein yield is much higher with bacterial expression, but solubility or folding problems can arise, preventing effective protein recovery. When the phosphorylation site in the substrate is known and specific antibodies against the phosphorylated form are available, immunoblotting can be performed to detect the activity of the kinase on the substrate [45]. Alternatively, the use of radiolabeled [ϒ-^32^P] ATP in the kinase reaction allows tracking of the transfer of radioisotope ^32^P to the kinase substrate by either radioactive scintillation methods or SDS-PAGE followed by autoradiography analysis [46]. This type of assay can be scaled up for global kinase substrate identification by using protein microarrays representing the whole proteome [47]. Both single and large-scale radioactive in vitro kinase assays have been effectively used to identify Slt2 substrates.

Although radioactive kinase assays are very sensitive, other detection methods are usually preferred to avoid potential adverse health effects. Among them, analog-sensitive (AS) kinase technology provides a very powerful tool for non-radioactive selective labeling of substrates in in vitro kinase assays [48]. In this approach, the active site of the kinase is engineered by mutating the normal gatekeeper to a smaller residue, such as glycine, to create a larger active site pocket that allows the enzyme to accept a bulky ATP analog, in which the γ-phosphate is replaced with a thiophosphate moiety. Then, proteins thiophosphorylated by AS kinase are reacted with the thiol-specific alkylating agent p-nitrobenzyl mesylate (PNBM) to generate a thiophosphate ester, which can be detected by immunoblotting with commercial thiophosphate ester-specific antibodies [49]. The advantage of this methodology is that it can be used to confirm direct phosphorylation by the engineered AS kinase of candidate substrates either purified or in complex samples, such as cell extracts. The bulky ATP analog is exclusively recognized by the modified AS kinase, which transfers the thiophosphate group to its target proteins in the sample. Therefore, only the labeled AS kinase substrates are detected by immunoblotting. The identity of the tagged substrate is then confirmed with tag-specific antibodies. This strategy has been successfully used to confirm several candidate targets of Slt2 [50].

The above-mentioned kinase assays are designed to be performed in vitro. Among the methods available for analyzing phosphorylation in vivo, a classical one is an electrophoretic mobility shift analysis, based on the change in band migration usually displayed by phosphorylated proteins. When antibodies or tagged versions of proteins are available, it is possible to detect such changes, which may reflect a modification in the phosphorylation status [51]. Eliminating the shift by phosphatase treatment confirms that it is caused by phosphorylation. If the shift is not clearly observable by conventional SDS-PAGE, phosphate-affinity technology (Phos-tag) can be used to induce slower migration of phosphoproteins by reversible binding of the Phos-tag reagent to phosphate moieties on them [52]. Candidate substrates of a given kinase can be identified by assessing the loss of protein phosphorylation in mutant cells lacking the active kinase. Mutagenesis of putative phosphorylation residues in the substrate leading to the loss of mobility shift allows identification of the actual phosphosites.

On a large-scale basis, MS-based phosphoproteomics enable global studies of dynamic protein phosphorylation in vivo [53]. Phosphoproteomic data, including phosphosite identification and phosphoprotein quantification, obtained under MAPK-stimulated vs. unstimulated conditions, or in mutants lacking the MAPK vs. wild-type cells, provide a map of potential kinase substrates. This strategy has been applied by our group to yeast cells overexpressing a constitutively active version of Pkc1 to unveil putative Slt2 targets [54].

Both in vitro and in vivo approaches have advantages and limitations. The former can provide substrate identification in a highly selective and sensitive manner, but the alteration of the stoichiometry of the reaction and the loss of cellular compartmentalization may result in the identification of false kinase substrates. In vivo assays can associate a kinase with authentic phosphorylation events, but direct phosphorylation cannot be inferred without additional experimentation [55]. Therefore, a combination of different strategies is required to designate a protein as a genuine MAPK substrate; therefore, it is the best way to obtain a complete and accurate picture of the MAPK-dependent signaling network.

## 3. Targeting Different Yeast Processes: Genuine Slt2 Substrates

Several lines of evidence from both in vivo and in vitro assays provide increased confidence to consider a protein as a kinase substrate. Thus, we selected as bona fide Slt2 substrates those proteins that have been found to be directly phosphorylated by Slt2 in vitro, but they have also been strongly linked to Slt2 in vivo, either genetically or biochemically, for example, by being phosphorylated under CWI pathway activating conditions and/or displaying a genetic or physical interaction with Slt2 (Table 1, Figure 1). In the next section of this review, we recapitulate the Slt2 substrates that meet these criteria and discuss the relevance of this phosphorylation to yeast physiology described to date. In addition, we include information on potential Slt2 targets that have not yet been fully validated as authentic substrates.

Although some non-catalytic roles have been described for Slt2, most of its functions are carried out through direct phosphorylation of protein targets. Even though Slt2 transiently localizes in polarity sites, such as the tip of emergent buds and the bud neck during cytokinesis, this MAPK is particularly enriched at the nucleus [56]. The predominant nuclear localization of Slt2 reflects its preeminent role in regulating gene expression through the phosphorylation of nuclear targets. However, as new Slt2 cytosolic substrates are discovered, the implication of this MAPK in the control of additional cellular processes, including the cell cycle and signaling pathways, becomes more evident (Figure 1).

### 3.1. Feedback Regulation of the CWI Pathway: Rom2, Mkk1/2, and Msg5

With the aim of ensuring the best fitness or even cell survival, Slt2 adjusts the signal flow through the CWI pathway by phosphorylating several of the components that constitute this signaling cascade. While some of these phosphorylation events enhance MAPK activation, others negatively regulate it. For example, under stress conditions that require full activation of the CWI pathway, Slt2 guarantees the induction of an appropriate adaptive cellular response by inhibiting the action of its main negative regulator, the phosphatase Msg5. However, when excessive activation of the CWI pathway is detrimental to the cell, Slt2 inhibits its own activation through a negative feedback loop acting on the GEF Rom2 and MAPK activating proteins Mkk1/2 (Table 1).

Rom2, Rom1, and Tus1 are the GEFs that activate the Rho1 GTPase in *S. cerevisiae,* acting upstream of the CWI MAPK module (Figure 1) [4,72]. Under normal growth conditions, basal activity of the CWI pathway is required to maintain the polarized distribution of actin and control cell wall synthesis [2]. In this situation, Rom2 localizes to the growing bud surface during bud emergence and to the bud neck during cytokinesis in a cell cycle-dependent manner [73,74], similarly to Rho1 [75]. However, in response to heat stress, which activates the CWI pathway, Rom2 becomes depolarized [12] and undergoes an Slt2-dependent mobility shift [25]. In vitro phosphorylation assays confirmed that Slt2 directly phosphorylates Rom2. This retrophosphorylation event would lead to the redistribution of Rom2 from the bud to the cell periphery and to subsequent inactivation of Rho1 activity, suggesting the existence of an Slt2-dependent feedback control that downregulates CWI pathway signaling by depriving Rho1 of its GEF when cells are exposed to adverse conditions [25]. Even though the exact position of this phosphorylation has not been described, several studies have revealed that Rom2 is phosphorylated at S171 and T216 by the CDK Cdc28 [76], and at five additional S/T-P sites (S126, S233, S391, T398, S494), which appear to be phosphorylated in several phosphoproteomic analyses, pointing to these sites as potential Slt2 targets (for details, see the *Saccharomyces* Genome Database at [77]).

Mkk1 and Mkk2 interact with Slt2 [78], leading to its phosphorylation at T190 and Y192 and its subsequent activation [79]. These MAPKKs were first described to be functionally redundant [80]. However, later studies attributed a preeminent role of Mkk1 over Mkk2 in the transmission of signals through the CWI pathway [9,16]. Specifically, it has been suggested that priming phosphorylation at Y192 is mainly carried out by Mkk1, and that this modification is necessary for subsequent phosphorylation at T190 [18]. On the other hand, Slt2 has been shown to phosphorylate Mkk1 and Mkk2 in vivo and in vitro by mobility shift and kinase assays, respectively [57]. In particular, Mkk2 retrophosphorylation at S50 does not affect its localization, stability, or ability to interact with Slt2, but it appears to downregulate its function, constituting a negative feedback regulatory mechanism of the CWI pathway [57]. Intriguingly, it has been demonstrated that, in contrast to the wild-type version of Slt2, the threonine monophosphorylatable mutant, Slt2^Y192F^, is able to retrophosphorylate Mkk1 but not Mkk2 [18], suggesting that this feedback phosphorylation mechanism can be different in each MAPKK, providing high versatility to this pathway. Retrophosphorylation of upstream components by MAPKs has also been shown to regulate signaling specificity and intensity in other yeast [81,82] and mammalian [83] MAPK pathways.

Msg5 is a dual-specificity phosphatase (DSP) that negatively regulates the mating and CWI MAPK pathways by dephosphorylating threonine and tyrosine residues located at the activation loop of Fus3 and Slt2, respectively [10]. Importantly, following heat stress, Msg5 is phosphorylated in vivo in an Slt2 kinase activity-dependent manner. Moreover, Slt2 directly phosphorylates the phosphatase-dead version Mgs5^C319A^ in in vitro kinase assays. It is likely that this phosphorylation negatively regulates the interaction between Msg5 and Slt2, and has been thus proposed to serve as a mechanism by which Slt2 ensures its proper phosphorylation state and the subsequent cell wall remodeling response, as long as cell surface stress is present [58]. Since Msg5 also negatively regulates Fus3, it has been suggested that retrophosphorylation of Msg5 could prevent it from acting on Slt2, but not Fus3, providing substrate specificity [58]. The precise residues phosphorylated by Slt2 have not yet been identified. Different phosphoproteomics studies have revealed that Msg5 contains 10 S/T-P phosphorylated sites, six of them in the N-terminal regulatory domain (S22, S62, S85, S115, S135, and T178) and four in the C-terminal half of the protein (S377, S422, T434, and T437). Although both domains are phosphorylated in vitro, only the latter seemed to be causative of the electrophoretic mobility shift displayed by Msg5 upon CWI activation [50].

Reciprocal regulation between MAPKs and DSPs is a conserved modulatory mechanism that is also found in mammalian cells [84,85,86]. Considering that Slt2 activation leads to a decrease in the overall amount of Msg5 [58], it is quite possible that Msg5 phosphorylation by Slt2 also negatively regulates the stability of this DSP, sustaining Slt2 activation by Msg5 degradation.

### 3.2. Slt2 Impinges on Central Yeast Signaling Pathways via Bcy1 and Avo2/3

Besides regulating its own pathway, Slt2 controls the activity of cAMP-dependent protein kinase (PKA) and target of rapamycin (TOR) complex 2 (TORC2) through phosphorylation of their subunits Bcy1 and Avo2/3, respectively (Table 1).

Bcy1 is the negative regulatory subunit of PKA [87]. Working together with the TOR complex 1 (TORC1) pathway, the PKA pathway regulates central processes for yeast growth, such as translation, ribosome biogenesis, autophagy, stress response, glucose metabolism, and life span [88]. In the absence of glucose, Bcy1 forms an inactive heterotetrameric complex composed by a Bcy1 dimer and two catalytic subunits, which are encoded by three homologs, *TPK1*, *TPK2*, and *TPK3*. However, in the presence of fermentable sugars, cAMP is synthetized, then it binds to Bcy1, causing its dissociation from the complex and the subsequent release of the Tpk active catalytic subunits that promote cell growth in these conditions [88,89].

The phosphorylation status of Bcy1 affects its affinity for catalytic subunits Tpk1–3 [90] and the localization of PKA. In yeast growing rapidly on glucose, PKA is almost exclusively localized in the nucleus. However, under stress conditions such as growth on a non-fermentable carbon source or an increase in temperature, Bcy1 is phosphorylated by kinases Yak1 and Mck1, causing its cytoplasmic localization via interaction with the protein Zds1 [91,92]. Bcy1 is also phosphorylated by Mck1 in response to DNA damage, restraining mitosis under these conditions [93]. In addition, inhibition of TORC1 with rapamycin leads to Bcy1 phosphorylation on several sites, including T129. This phosphorylation is totally abolished in cells lacking *SLT2*, and recombinant Bcy1 is phosphorylated in vitro on T129 by Slt2 purified from rapamycin-treated cells, indicating that Slt2 directly phosphorylates Bcy1. Upon rapamycin-dependent TORC1 inhibition, Sch9 becomes inactivated, promoting Slt2 hyperphosphorylation in vivo. This allows the subsequent Slt2-mediated phosphorylation of Bcy1 and the resulting inhibition of PKA catalytic activity. However, it is important to note that Bcy1 T129 phosphorylation is not exclusively dependent on TORC1 inhibition, but is also induced in vivo under different stresses, leading to activation of the CWI pathway, such as by cell wall disrupting agents or a genetically activated Bck1 version [28]. Strikingly, T129 is not a canonical proline-directed MAPK phosphosite, but rather a TS site. Considering that Bcy1 contains a TP site at T131 and that phosphoproteomic analyses have revealed that this residue is phosphorylated [77]), it is possible that T131 phosphoresidue participates in T129 recognition by Slt2.

The evolutionarily conserved TORC2 complex is an essential regulator of plasma membrane homeostasis in *S. cerevisiae.* TORC2 contains four essential core subunits (Avo1, Avo3, Lst8, and Tor2), two classes of peripherally located non-essential subunits (Avo2 and Bit61 or its paralog Bit2), and two essential ancillary subunits (Slm1 and Slm2) that shuttle from eisosomes to TORC2 following plasma membrane stress [94]. Phosphoproteomic analyses have detected in vivo phosphorylation of the MAPK S/T-P target motifs in all proteins comprising TORC2 except Bit2 [77].

Particularly, Avo2 and Avo3 are phosphorylated at several sites in vivo upon overexpression of a constitutively active version of Pkc1, which leads to the activation of Slt2 and Hog1 MAPKs. However, Avo2 hyperphosphorylation was markedly reduced in *slt2*Δ cells but not in *hog1*Δ cells, as detected by Phos-tag SDS-PAGE, confirming that Slt2 is the major MAPK responsible for this phosphorylation. In addition, Avo2 and the N-terminal fragment of Avo3 (1–100), which contain nine and six S/T-P sites, respectively, are robustly phosphorylated in vitro by Slt2. Mutation of these residues to non-phosphorylatable alanine eliminates and markedly reduces Avo2^9A^ and Avo3(1–100)^6A^ Slt2-dependent phosphorylation, respectively, confirming that both proteins are direct targets of Slt2 [29]. Slt2-dependent phosphorylation of Avo2 downregulates TORC2 activity on its primary downstream effector, Ypk1. Because the expression of the phosphomimetic Avo2^9E^ version renders cells sensitive to myriocin-induced sphingolipid depletion, showing significant displacement from the plasma membrane, it has been proposed that phosphorylation of Avo2 by Slt2 promotes its dissociation from TORC2 [29]. Thus, the Slt2-mediated phosphorylation of Avo2 constitutes the first evidence that an MAPK pathway regulates TORC2 function and reveals the regulatory circuitry by which *S. cerevisiae* controls the growth-promoting functions of TORC2 depending on cell wall stress.

### 3.3. Cell Wall Stress-Related Gene Transcription: Rlm1 and SBF Complex

In addition to its important role in regulating signaling events, the main function of Slt2 is to regulate transcription factors, with the aim of adjusting the transcriptional response to the environmental context [95]. CWI activation triggers the expression of a characteristic pattern of stress-related genes that allows the yeast to cope with cell wall or plasma membrane insults. This transcriptional program is regulated by Slt2 through the phosphorylation of two transcription factors, Rlm1 and SBF (Table 1) [96].

Rlm1 was the first identified Slt2 substrate, and its ability to rescue the growth inhibition caused by overexpression of a hyperactive version of Mkk1 [43] prompted additional investigations. Rlm1 drives the main CWI transcriptional reprogramming response to cell wall stress [96]. Hence, upon CWI activation, a particular cluster of genes is induced, including *SLT2* and *RLM1* [97]. This way, Rlm1 phosphorylation by Slt2 leads to a positive feedback circuit that ensures a high signaling flow through the pathway [26].

In vitro phosphorylation assays indicated that upon heat stress, Slt2 phosphorylates a fragment of Rlm1 comprising amino acids 329 to 445, containing three S/T-P motifs, S374, S427, and S439 [44]. A later study demonstrated that S427 and S439 residues are responsible for the majority of the transcriptional activation function of Rlm1 [98]. Neither of these two residues appears as phosphorylated in a phosphoproteomic analysis [77]. Further studies showed that the triple *rlm1* mutant in the aforementioned sites is still phosphorylated by Slt2 and retains transcriptional activity. Mutation of the seven additional S/T-P sites of Rlm1 outside of the DNA-binding domain, S234, S261, T276, S299, S518, T646, and T654, totally abolishes the Slt2-dependent phosphorylation of Rlm1 and its transcriptional activity, indicating that together with S374, S427, and S439, other residues of Rlm1 are phosphorylated by Slt2 and required for full transcriptional activation [99]. In vitro kinase assays with individual non-phosphorylatable Rlm1 mutants in these positions would make it possible to identify all the regulatory phosphorylation sites and to clarify the mechanism underlying Slt2-dependent Rlm1 activation.

Besides phosphorylation, activation by Slt2 requires the integrity of a MAPK docking site in Rlm1 [98]. However, a recent study demonstrated that the lack of a functional CD domain in Slt2 does not completely abolish signaling to Rlm1, suggesting that additional sites are involved in the interaction between Slt2 and Rlm1 [18]. This study also showed that Rlm1 phosphorylation depends on the presence of both T190 and Y192 residues within the activation loop of Slt2, since monophosphorylatable mutants are as ineffective as the catalytically inactive Slt2 version inactivating Rlm1 [18].

In a less prominent position than Rlm1, the CWI pathway also organizes its adaptive transcriptional response through activation of the SBF complex. SBF and MBF are transcription factor complexes that regulate the activation of the transcriptional program that mediates the G1/S transition [100]. SBF is a heterodimeric protein composed of DNA binding factor Swi4 and transcriptional activator Swi6. The interaction of Swi6 with Swi4 relieves an autoinhibitory intramolecular association of the Swi4 C-terminus with its DNA binding domain, allowing binding to its target promoters [101]. Although the CWI pathway has been related to the SBF transcriptional complex at multiple levels, its role in the regulation of the G1/S transcriptional program remains to be fully elucidated [21]. However, the role of SBF in regulating cell wall stress-induced gene transcription is better understood. It was demonstrated that overexpression of *SWI4* restored the viability of *slt2*Δ cells exposed to cell wall damage, and that SBF controlled the transcription of several cell wall-related genes, linking this complex to Slt2 function [102]. Although in vitro phosphorylation of Swi4 by Slt2 was reported quite some time ago [60], subsequent experiments showed that Slt2 activates SBF by a mechanism in which its kinase activity is dispensable. Slt2 relieves autoinhibitory Swi4 interaction by binding to an MAPK docking site near the C-terminal Swi6 binding site, leading to the association of Swi4 with the promoter region of a subset of genes (*FKS2, CHA1, YLR042C, YKR031w*) to enhance their expression upon cell wall stress [30,31,103].

On the other hand, in vivo and in vitro phosphorylation assays have demonstrated that Slt2 directly phosphorylates Swi6 upon heat shock. However, Swi6 is initially directed to the promoters of its stress-related target genes, where it associates with Swi4 and Slt2 for transcription to initiate in a Slt2-catalytic activity independent manner. It is the phosphorylation of Swi6 by Slt2 on Ser238 that subsequently drives this transcription factor out of the nucleus, since this modification interferes with the function of an adjacent nuclear localization signal (NLS). By this means, Slt2 exerts a negative regulation on the transcriptional function of Swi6 [61]. Given that Swi6 acts as a transcriptional activator, it is not clear whether the Slt2-mediated phosphorylation of Swi6 could affect the expression of the other target genes it regulates. In addition, the Slt2-mediated cytoplasmic re-localization of Swi6 could promote its association with other proteins.

### 3.4. Regulation of RNA Polymerase Holoenzyme Complex: Cyclin C, Med13, and Rbp1

Along with activating transcription factors Rlm1 and SBF, Slt2 also modulates gene expression by directly controlling the transcription machinery. Three components of the RNA polymerase holoenzyme complex are phosphorylated by Slt2 upon stress conditions: the cyclin C (Ssn8), the Mediator component Med13 (Ssn2), and the RNA polymerase II (Pol II) catalytic subunit (Rpb1) (Table 1). Pol II is a complex 12-subunit enzyme responsible for mRNA transcription in eukaryotes. Despite its complexity, Pol II itself lacks the ability to initiate transcription and needs to interact with different proteins and complexes to regulate its activity. For example, the association of Pol II with the Mediator is required for the initiation of transcription of some eukaryotic genes [104] and the repression of a subset of others [105]. The Mediator is a multi-subunit transcriptional coactivator complex of proteins, highly conserved among eukaryotes. Structurally, Mediator proteins are assembled separately into a core Mediator and a dissociable subcomplex called Cdk8 kinase module (CKM), containing Med12-Med13-Cdk8-Ssn8 [106]. The CKM plays an important function as a transcriptional repressor by modulating the activity of Rpb1 through phosphorylation of its carboxy-terminal domain (CTD) [107].

Yeast Ssn8 is generally degraded upon exposure to some stresses, which relieves the transcriptional machinery from its repression function and allows stress-induced gene expression [108,109]. Particularly, exposure to reactive oxygen species (ROS) promotes the Slt2-dependent phosphorylation of Ssn8 in the only MAPK target site S266, and subsequent proteasomal degradation. Interestingly, this is a specific role for Slt2 in response to ROS, but not to other types of cellular stress, such as thermal stress [62,63,110,111]. Upon Slt2 phosphorylation, Ssn8 translocates from the nucleus to the cytoplasm, where it promotes mitochondrial fission via a transcription-independent mechanism and induces programmed cell death before its complete degradation. In addition, the Mediator component Med13 is also degraded by the proteasome in response to ROS. This is a sequential process, in which the first step consists of priming phosphorylation by Cdk8-Ssn8, and the second step involves Slt2-mediated phosphorylation on S266 of Ssn8, facilitating Slt2-dependent Med13 phosphorylation, which in turn leads to its degradation [64]. This parallel regulation of two different targets within the same complex ensures Ssn8 nuclear release and subsequent activation of transcriptional and mitochondrial responses when facing oxidative stress, to finally promote cell survival.

Rpb1 CTD is subjected to extensive regulatory phosphorylation not only by the Ssn8-Cdk8 complex, as mentioned above, but also by many other protein kinases. Usually, these modifications are carried out in the CTD consensus repeated heptad Y_1_S_2_P_3_T_4_S_5_P_6_S_7_. Even though the most common modifications take place in S2 and S5, Y1 phosphorylation has been shown to play a role in the regulation of gene expression. Slt2 is the kinase responsible for Rbp1 Y1 phosphorylation, and this event is important for upregulating the transcription of stress-induced genes, especially those related to cell wall stress, iron homeostasis, and processes related to oxide-reductive stress. Rbp1 Y1 phosphorylation by Slt2 seems to control the function of the Nrd1-Nab3-Sen1 (NSS) complex during the stress response, preventing this transcription termination complex from prematurely binding to the CTD [65]. So far, this is the only reported tyrosine phosphorylation executed by Slt2, except for the auto-phosphorylation on its TEY activation domain after removal of the C-terminal tail [112]. It is important to note that a non-phosphorylatable Rpb1 Y1F mutant exhibits increased Ssn8-Cdk8 promoter occupancy on several stress genes. Given the fact that Ssn8-Cdk8 is a transcriptional repressor, Slt2-mediated Rbp1 Y1 phosphorylation may facilitate Ssn8-Cdk8 recognition by Slt2, favoring stress gene expression.

### 3.5. Epigenetic Control of Gene Expression and Yeast Life Span Extension: Sir3 Phosphorylation

Chromatin consists of packaged genomic DNA interacting with histones. The dynamic control of chromatin is an important layer in the regulation of gene expression. Besides the direct modification of DNA and histones, remodeling protein complexes play a key role in chromatin regulation [113]. The silent information regulator (SIR) complex, which consists of Sir2, Sir3, and Sir4, is involved in gene silencing on mating-type loci *HML* and *HMR* on chromosome III and regions surrounding the telomeres [114,115].

Previous evidence indicated that Sir3 is a phosphoprotein that becomes hyperphosphorylated upon exposure to several stresses [116]. Further work carried out independently by two groups confirmed, by in vitro kinase assays, that Sir3 is a direct substrate of Slt2 (Table 1) [66,67]. Slt2 phosphorylates Sir3 upon rapamycin and chlorpromazine treatment, but not in nutrient starvation conditions [66]. This phosphorylation event impedes Sir3 from exerting its silencing function on subtelomeric regions and causes the derepression of certain cell wall stress-related genes [66], such as seripauperin (*PAU*) genes, which are located at these sites [117]. Sir3 phosphorylation by Slt2 therefore constitutes an additional mechanism by which the CWI MAPK pathway acts as an enzymatic regulator of gene expression. Mutants affected in cohesin, a key architectural chromosomal protein complex, have been shown to display telomere silencing defects as well as Slt2-dependent Sir3 hyperphosphorylation. However, in this case, Slt2 activity contributed to derepression only to a very limited extent, suggesting the existence of a Sir-independent mode of repression mediated by cohesin [118]. Several studies with different organism models, including *S. cerevisiae*, have shown that sirtuins are linked to aging [119]. Given that Sir3 is required for the recruitment of sirtuin Sir2 to telomeres [120], and that Sir2 activity has been linked to telomere silencing and a subsequent increase in life span [121], Sir3 phosphorylation by Slt2 could promote dissociation of Sir2 from telomeres, with an impact on life span and aging in *S. cerevisiae.* Indeed, Sir3 phosphorylation on S275 and S282 by Slt2 has been shown to shorten life span [67].

### 3.6. Regulation of mRNA Nuclear Export: Nab2

Restriction of the mRNA nuclear exit constitutes an important mechanism by which eukaryotic cells control protein expression by preventing transcripts from entering the cytoplasm in stress situations. In *S. cerevisiae*, exporting of mRNA is blocked when defects in splicing are detected [122] or in stress circumstances, such as heat stress [123]. However, there is also selective exporting of transcripts that is induced under such stress conditions, so that only those transcripts required in a particular context are exported to the cytoplasm. For example, heat shock protein transcripts are selectively exported from the nucleus in response to heat stress [123]. The selective mRNA nuclear export is controlled by post-translational modifications of export adaptors, which dissociate them from mRNA [124].

The CWI MAPK Slt2 controls the export of transcripts through phosphorylation of the polyadenylated RNA-binding protein Nab2, which mediates the nuclear mRNA export. Nab2 is phosphorylated upon heat shock, displaying an Slt2-dependent mobility shift. In vitro kinase assays have demonstrated that Slt2 directly phosphorylates Nab2 on residues T178 and S180 (Table 1) [68]. Moreover, the phosphorylation of bacterially produced Nab2 by an AS version of Slt2 (Slt2-AS) has been observed in our laboratory [50]. The Slt2-dependent phosphorylation of Nab2 reduces its binding to the export receptor Mex67 [68]. In this work, a model was proposed in which activated Slt2 promoted the nuclear retention of non-heat-shock mRNAs by uncoupling Mex67 from Nab2 and possibly other components of mRNA export machinery during heat shock. Coincident with Nab2 phosphorylation, this protein and the mRNA binding protein Yra1 co-localize in nuclear foci with the nuclear pore-associated myosin-like protein 1 (Mlp1), a protein involved in mRNA retention. However, Nab2 nuclear focus formation and Nab2 phosphorylation are independent, suggesting that several mechanisms are implicated in mRNA transport during heat stress. In addition to mRNA nuclear export, Nab2 has been implicated in the previous proper 3′ end processing as well as splicing of nascent RNA, serving as a checkpoint for the fidelity of pre-mRNA processing [122,125]. Thus, it is tempting to speculate that Slt2 may also be implicated in the regulation of these processes through Nab2 phosphorylation.

### 3.7. Control of Cell Cycle: Sic1 and Mrc1

MAPKs are key players in the control of the cell cycle in eukaryotes. In response to plasma membrane and cell wall damage, the CWI pathway regulates cell cycle progression at different points [21]. In line with this, the Slt2-dependent phosphorylation of Sic1 and Mrc1 (Table 1) has been implicated in cell cycle arrest at Start point and S-phase, respectively.

When the Start transcriptional program is activated, the cyclin dependent kinase inhibitor (CKI) Sic1 is sequentially phosphorylated on multiple sites within its N-terminal region, first by the G1/S CDK–cyclin complexes Cln1,2-Cdc28, and later by Clb-Cdc28. These phosphorylation events target Sic1 for degradation mediated by the ubiquitin ligase SCF^Cdc4^, thus allowing progression from the G1 to S phase. TORC1 inhibition by rapamycin treatment or nitrogen limitation promotes the activation of both Greatwall/Rim15 kinase and Slt2. As a result, whereas Slt2 phosphorylates Sic1 in T173, Rim15 inhibits the phosphatase PP2A-Cdc55, which dephosphorylates Sic1 in this site. These events greatly stabilize Sic1, since T173 phosphorylation is critical for Sic1 stability in rapamycin-treated cells [126] by preventing its association with SCF^Cdc4^ [127], thus avoiding Sic1 degradation. Consequently, the stabilized pool of Sic1 inhibits the G1 to S transition, and thus promotes cell cycle arrest in a stress context [128]. Such Slt2-dependent Sic1 phosphorylation at T173 has been demonstrated both in vitro and in vivo (Table 1). Even though this phosphorylation has been well characterized in rapamycin-treated cells, an increase in Sic1 T173 phosphorylation is also observed during the G1 phase in proliferating cells [128]. Sic1 is also stabilized after SDS-induced plasma membrane damage [129], a condition that triggers Slt2 activation. Thus, Slt2 may restrain cell cycle progression through Sic1 stabilization in other situations that have not yet been studied.

Another substrate through which Slt2 exerts cell cycle control is Mrc1, a component of the DNA replication complex. Mrc1, an evolutionarily conserved replisome-associated factor required for efficient DNA replication [130], couples DNA polymerase and helicase activities [131] and is involved in establishing the S-phase checkpoint to prevent genome instability [132]. Upon heat shock, Mrc1 is phosphorylated in its N-terminus at positions T169, S215, and S229 by the MAPK Slt2 (Table 1), causing a delay in DNA replication and favoring widespread transcriptional reprogramming. This way, Slt2 prevents transcription–replication conflicts that may arise in situations in which a massive transcriptional response and DNA replication occur simultaneously [69]. A recent study showed that a sublethal concentration of ethanol causes DNA replication stress and relocalization of Mrc1 from the replication fork to a perinuclear compartment, affecting replisome stability, replication rate, and genome stability. However, whether ethanol triggers Mrc1 phosphorylation is still unknown [133]. Future studies analyzing the effect of Slt2-dependent phosphorylation on Mrc1 localization will help to elucidate the mechanism underlying this S-phase checkpoint.

Interestingly, both Sic1 and Mrc1 are phosphorylated on the same sites by Hog1, the MAPK of the yeast osmolarity pathway [127,134]. This suggests that these proteins contain regulatory sites sensitive to changes in the environment, reflecting the variety of mechanisms that operate in cell cycle control.

### 3.8. Orphan Slt2 Substrates: Caf20, Rcn2, and Gga1

Several Slt2 substrates have been found in proteomic studies and confirmed through direct kinase assays, but the function of Slt2-exerted phosphorylation remains to be discovered.

As mentioned above, our group performed a phosphoproteomic analysis of *S. cerevisiae* upon overexpression of a constitutively active version of Pkc1, which led to hyperactivation of the CWI pathway. This assay generated a list of potential Slt2 target proteins, which contained peptides that appeared hyperphosphorylated at S/T-P sites in this condition [54]. Further experiments with Slt2-AS led us to confirm that among these candidates, Caf20, Rcn2, and Gga1 were direct targets of Slt2 phosphorylation in vitro (Table 1) [50].

The translation initiation repressor Caf20 is phosphorylated by Slt2 on the only consensus S/T-P site at T102 [50]. This residue has also been found to be phosphorylated in other phosphoproteomic studies [77]. Since Caf20 represses the translation of its target mRNAs, and mRNAs linked to processes such as cell cycle, intracellular signaling cascades, and cell morphogenesis were found to be enriched in the pool of Caf20-associated mRNAs [135], it is very possible that the CWI pathway specifically regulates the translation of these subsets of proteins through phosphorylation of this protein. 

The calcium/calmodulin-dependent protein phosphatase regulator Rcn2 [136] was phosphorylated by Slt2 on three target sites: S152, S160, and S255 [50]. Phosphorylation of this protein by Slt2-AS was totally lost only in the mutant protein with all three residues substituted by alanine. Each individual mutation resulted in a reduced phosphorylation, but the most intense effect was observed when S255, the only one of these Rcn2 phosphosites detected as hyperphosphorylated in our previous phosphoproteomic analysis [54], was mutated (Table 1). These three residues also appeared to be phosphorylated in a global phosphoproteomic analysis using the endoplasmic reticulum (ER) stress-inducing agent DTT [137]. Taking into account that calcineurin has an essential role in response to ER stress [138], and that DTT triggers activation of the CWI pathway [15,139], Slt2 phosphorylation of Rcn2 may be part of a regulatory mechanism by which the CWI pathway modulates the calcineurin response to ER stress.

The Golgi-associated protein Gga1, involved in protein trafficking, contains four S/T-P target sites, which have been shown to be phosphorylated in a different phosphoproteomic analysis [77]. Although the specific Slt2-dependent phosphoresidue remains undetermined, the strong negative genetic interaction found between *SLT2* and either *GGA1* or its paralog *GGA2* under cell wall stress conditions suggests a connection of the CWI pathway with trafficking from the Golgi complex to the vacuole [50].

### 3.9. Is There a Specific Slt2 Phosphorylation Signature?

**Figure 2:**
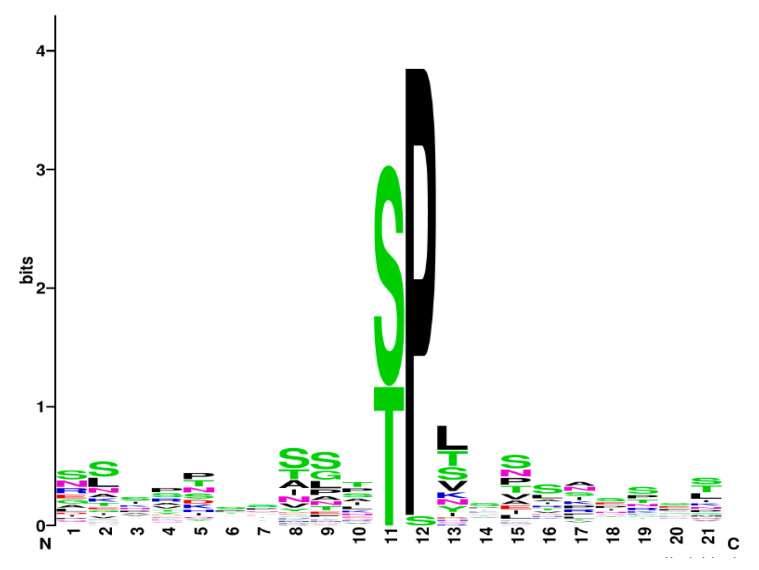
Slt2 consensus phosphorylation site. Motif logo representing Slt2 phosphorylation signature, obtained with WebLogo, a program for alignment and motif enrichment [140]. The 11th position at the logo corresponds to serine or threonine phosphorylated by Slt2, and the rest to 10 upstream/downstream amino acids surrounding this position. Complete list of sequences corresponding to phosphorylated peptides can be found in Table S1 and correspond to proteins included in Table 1 whose S/T phosphorylation sites are known.

In searching for an Slt2 consensus signature, we compared the S/T phosphorylation motifs of the 14 genuine Slt2 targets whose phosphorylation sites are known (Table 1, Table S1). We excluded Rpb1 because it is atypically hyperphosphorylated at tyrosine. As shown in Figure 2, most of the peptides analyzed are phosphorylated in the consensus MAPK site S/T-P [32], except Bcy1 which shows serine in the +1 position instead of proline. The expected Slt2 preference for an R at the P−3 position [34] was not found within the Slt2 phosphorylation consensus signature. Our analysis reveals the difficulties in establishing the rules that determine Slt2 specificity and limits the feasibility of predicting its phosphorylation sites. It is also interesting to note the importance of knowing the precise biological relevance of Slt2-mediated phosphorylation. However, while this is easily achieved in cases of single phosphorylation sites, the existence of multiple phosphorylation sites, which is common among Slt2 substrates, hinders this task.

## 4. Candidate Slt2 Substrates: A Growing List

Apart from the proteins listed above, which we have considered to be genuine Slt2 substrates, other proteins have been described that are phosphorylated in vitro by Slt2, or in vivo upon CWI-activating conditions, suggesting that they are likely Slt2 targets. They have been found through either systematic approaches, such as protein array screenings and phosphoproteomics, or targeted experiments. However, they do not meet the criteria initially indicated and more evidence is needed to consider them as bona fide Slt2 substrates. They are briefly reviewed below.

A global analysis of yeast protein phosphorylation by in vitro kinase assays performed with 87 kinases over a microarray representing the yeast proteome found direct phosphorylation of Slt2 on Brx1 and Cmk2 [141]. In spite of this, no further work has confirmed either these observations by individual in vitro or in vivo assays, or the precise phosphorylated positions. Brx1 is a ribosomal assembly factor [142] that contains a unique MAPK target site (S244), which was found to be phosphorylated in phosphoproteomic analyses [77]. Therefore, S244 is a very plausible target site for Slt2 on Brx1. On the other hand, Cmk2 is a calmodulin-dependent protein kinase that acts as a negative feedback controller within the calcium/calmodulin signaling pathway [143]. Although Cmk2 contains two MAPK target sites, neither of them has been reported to be phosphorylated [77].

Eisosome, also known as the membrane compartment of Can1 (MCC), is a protein complex distributed throughout the plasma membrane of *S. cerevisiae*. Eisosome formation is promoted by two paralogous proteins, Pil1 and Lsp1 [144]. Our quantitative phosphoproteomic analysis of yeast cells under Pkc1 hyperactivation revealed that Pil1 and Lsp1 displayed increased phosphorylation at MAPK target motif T233. Both proteins showed electrophoretic mobility promotion corresponding to their phosphorylation status, which was dependent on the presence of Slt2 in yeast cells [54]. Interestingly Pil1 T233 residue has also been described to be phosphorylated by Pkh1 and Pkh2 [145,146], the two redundant PDK1 homologs required for Pkc1 activation [7]. Considering that the sequence surrounding the T233 phosphorylation site in Pil1 does not match the consensus site for PDK kinases [147] and that Slt2 is downstream of Pkh1 and Pkh2 [2], T233 may be targeted by Slt2, thus regulating eisosome assembly and organization. However, we could not detect direct kinase activity on Pil1 by the Slt2-AS-based kinase assay in vitro [50], pointing to the possibility that an Slt2-dependent intermediate kinase is responsible for Pil1 T233 phosphorylation. In any case, the functional implication of Pil1 and Lsp1 phosphorylation remains controversial, given that Pil1 phosphorylation has been shown to promote eisosome assembly or disassembly depending on the phosphorylated residue [148].

Slt2 is mainly localized in the nucleus, where it controls, among other processes, transcription, epigenetic modification, and mRNA nuclear export through phosphorylation of the different nuclear substrates described above. In addition, this MAPK also localizes at the tip of small buds and at the mother-bud neck region in late mitosis, promoting the expansion of the daughter cell by new cell wall synthesis and stimulating septum construction for cell separation, respectively. Thus, even though no direct substrates at sites of polarized growth have been found, it is plausible that Slt2 phosphorylates proteins implicated in the regulation of these morphogenetic events. Among them, Bni4, a phosphoprotein with an important role in septum formation during cytokinesis [149], is a potential substrate of Slt2 within the yeast bud neck. In favor of this idea, it has been shown that Slt2 physically interacts with Bni4 and regulates its localization. Moreover, Bni4 contains several S/T-P sites with important roles in its function at the bud neck, and *slt2*Δ mutants exhibit a decrease in Bni4 phosphorylation [150]. An interesting phosphoproteomic analysis of the transcriptional response to the ER stressor DTT revealed Mkk1/2-dependent phosphorylation of 28 proteins, mainly involved in budding, polarity, cytoskeleton, and endocytosis (Spa2, Exo84, Bps1, Kin1, Mon2, Myo5, Vrp1, Yck1, Bbc1, Sec31, Vrp1, Twf1, and Bud6), which uncovers new potential Slt2 substrates related to these functions. Because the vast majority of phosphorylation was observed in long-term treatment with DTT, these protein modifications may be involved in the adaptive response to DTT-induced ER stress [137].

As mentioned above, a large-scale phosphoproteomic analysis under conditions that lead to activation of the CWI pathway has yielded potential Slt2 substrates that remain to be individually assayed. A recent example of this approach is the rapamycin-induced phosphoproteome, which has shown the Slt2-dependent phosphorylation of seven proteins, including the transcriptional repressor Mig1 and the calcineurin-activated transcription factor Crz1 [151]. The Slt2-dependent phosphorylation of Cmk2, Rcn2, and Crz1, all proteins related to calcium homeostasis [136,143], together with the reported negative epistatic interactions between Slt2 and calcineurin [152], provide evidence of the involvement of the MAPK Slt2 in the control of the calcium/calcineurin signaling pathway.

## 5. Concluding Remarks

**Figure 3:**
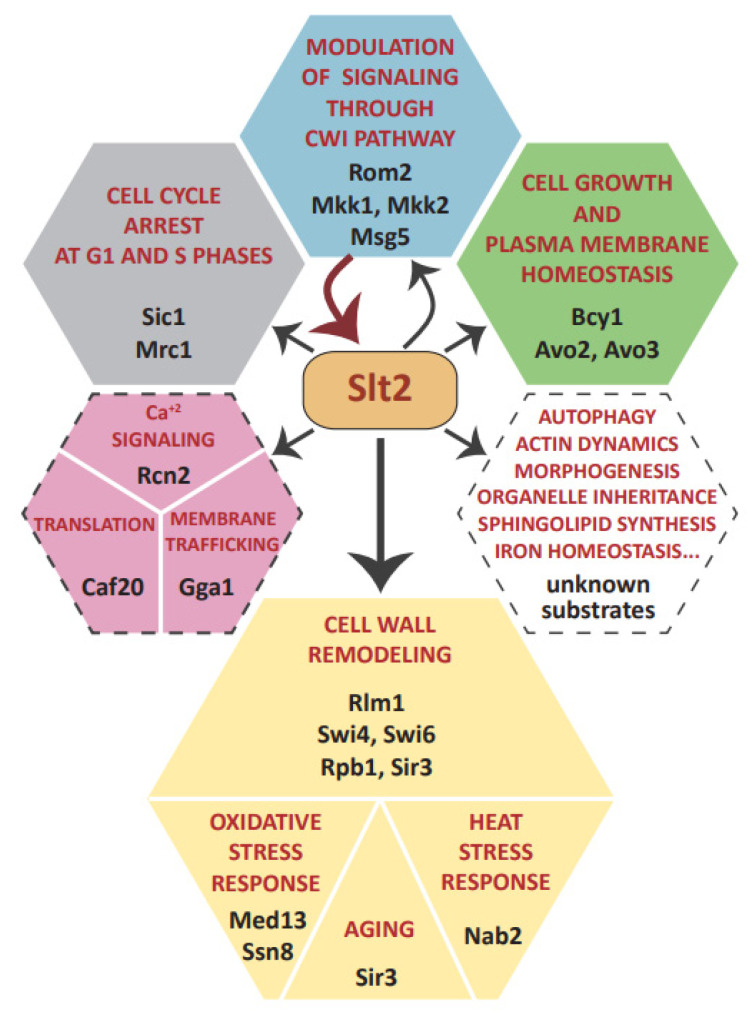
Graphical scheme showing cellular processes that are affected by Slt2 through the phosphorylation of the indicated substrates. Cellular processes are grouped by color based on the functional implication of substrate phosphorylation, as in Table 1: modulation of signaling through CWI pathway (blue), regulation of other signaling pathways (green), regulation of gene transcription and mRNA transport (yellow), cell cycle control (gray), and undetermined Slt2-dependent phosphorylation role (pink). The hexagons delimited by dashed lines show suggested but not formally proven Slt2-dependent functions (pink), or Slt2-regulated processes for which the specific substrates involved have not yet been identified (white).

Work over the last 30 years has provided evidence that Slt2, the MAPK of the CWI pathway, not only plays a key role in cell wall remodeling, but also has an important function in the control of cell signaling through its own and other pathways, and in coordinating essential physiological processes such as cell cycle, morphogenesis, and responses to different stress situations (Figure 3). The mechanism of action of Slt2 goes beyond its role as a protein kinase, as it has been shown to have kinase-independent functions. However, most of its wide cellular effects rely on its kinase activity. Thus, it is expected that the number of Slt2 substrates is much larger than that found to date. Furthermore, only a few of the already described substrates have been thoroughly characterized.

Exhaustive exploitation of genetic screening and proteomic approaches will reveal a good number of novel putative substrates of Slt2 in the near future. These efforts should further include biological validation experiments with the large number of tools available for the rapid confirmation of genuine Slt2 substrates, including hyperactive, kinase-dead, and analog-sensitive versions of this kinase [18,50]. The biological interpretation of the data should bring important information to link signaling through the CWI MAPK module to Slt2 functional roles.

Many currently known substrates of Slt2 are transcription factors or proteins related to transcriptional regulation, which underlines both the preeminent role of the CWI pathway as a regulator of gene expression under stress conditions and the induction of specific mRNAs as a hallmark of the response to cell wall insults. However, there is an important gap in the knowledge regarding other outputs of this key MAPK. For example, it would be important to unveil the unknown substrates responsible for key functions of Slt2, such as autophagy and inheritance of organelles, the control of sphingolipid synthesis, and actin cytoskeleton dynamics, among others (Figure 3). In addition, there are still some interesting issues to be addressed. For example, keeping in mind that Slt2 is transiently localized in polarity sites, it is surprising that no genuine substrate has been identified at this cellular localization. Additionally, as occurs with other MAPKs, such as ERK1/2, Slt2 dimerizes [153]. While it has been shown that dimerization impacts the activation of different pools of ERK substrates [154], the role of dimerization on Slt2 functionality and substrate targeting is still unknown.

In sum, over the coming years, new insights into how Slt2 shapes yeast cell integrity are likely to emerge. Identifying new Slt2 substrates will also help us to understand how pathogenic fungi use this widely conserved CWI pathway for virulence and adaptation to antifungal-induced stress. Thus, we should keep our eyes wide open, as this important new information could translate into applied medical research.

## Figures and Tables

**Table 1:** Genuine Slt2 substrates, indicating in vitro and in vivo phosphorylation assays used, physiological role and consequences of Slt2-dependent phosphorylation, and precise phosphosites. Substrates are grouped by color based on their functional implication: modulation of signaling through CWI pathway (blue), regulation of other signaling pathways (green), regulation of gene transcription and mRNA transport (yellow), cell cycle control (gray), and undetermined Slt2-dependent phosphorylation role (pink).

Substrate	In Vitro Kinase Assay			In Vivo Kinase Assay		Effects on Protein Function/Cell Physiology	P-Site	Ref.
	Method	Stimulus ^a^	Substrate Expressed in	Method ^b^	Stimulus ^a^			
**Rom2**	Radioactive	Heat stress	Yeast	Mobility shift	Heat stress	Cellular redistribution/ Inactivation of Rho1-mediated CWI signaling	ND	[25]
**Mkk1**	Radioactive AS kinase	Heat stress	Bacteria	Mobility shift	Vanadate	Downregulation /CWI signaling attenuation	ND	[50,57]
**Mkk2**	Radioactive	Heat stress	Bacteria	Mobility shift	Vanadate	Downregulation /CWI signaling attenuation	S^50^	[57]
**Msg5**	Radioactive AS kinase	Heat stress	Bacteria **^c^**	Mobility shift	Heat stress Cell wall stress (CFW)	Reduced binding to Slt2 /Increased Slt2 activation	ND	[50,58]
**Bcy1**	Radioactive	Rapa	Bacteria	Anti- p-Bcy1 (T^129^)	Rapa, SDS, CR, vanadate, Bck1 **^d^**	PKA inhibition	T^129^	[28]
**Avo2**	Radioactive	Pkc1 **^e^**	Bacteria	Phos-tag	Pkc1 **^e^**	Negative regulation of TORC2 activity	(T^157^, T^232^, S^246^, S^253^, S^262^, T^323^, S^328^, T^343^, S^348^) **^f^**	[29]
**Avo3**	Radioactive	Pkc1 **^e^**	Bacteria **^g^**	Phos-tag **^h^**	Pkc1 **^e^**	ND	(S^12^, T^25^, T^29^, S^51^, S^85^, S^88^) **^f^**	[29]
**Rlm1**	Radioactive	Heat stress	Bacteria **^g^**	Mobility shift	Heat stress	Activation/ Transcriptional induction of cell wall genes	S^374^, S^427^, T^439^ (S^234^, S^261^, T^276^, S^299^, S^518^, T^646^, T^654^) **^f^**	[44,59]
**Swi6**	Radioactive	Heat stress	Bacteria	Mobility shift	Heat stress	Nuclear exit/Downregulation of transcriptional activity	S^238^	[60,61]
**Swi4**	Radioactive	Heat stress	Insect cells	Epistatic and physical interaction with Slt2	Heat stress	ND	ND	[60]
**Ssn8** **(Cyclin C)**	Radioactive	Oxidative stress (H_2_O_2_)	Bacteria	Epistatic and physical interaction with Slt2	Oxidative stress (H_2_0_2_)	Cytoplasmic release, mitochondrial targeting and degradation/ Mitochondrial fission and transcriptional activation of stress genes	S^266^	[62,63]
**Med13**	Radioactive	Vanadate	Bacteria **^g^**	Epistatic interaction with Slt2	Oxidative stress (H_2_0_2_)	Degradation/ Cytoplasmic release of cyclin C	T^835^, T^837^	[64]
**Rpb1**	Anti-p-Y	Heat stress	Bacteria **^g^**	Anti-p-Y	Cell wall stress (CFW)	Activation/ Transcriptional induction of stress genes	Y_1_ of heptad repeats YSPTSPS	[65]
**Sir3**	Radioactive	Rapa, Heat stress	Bacteria, Bacteria **^g^**	Mobility shift	Rapa, Non- stimulated cells	Reduced association with subtelomeric sequences/ Derepression of *PAU* genes, Chronological lifespanshortening	S^275^, S^282^ S^289^, S^295^	[66,67]
**Nab2**	Radioactive	Non- stimulated	Bacteria	Mobility shift	Heat stress	Non-hsp RNA retention in the nucleus/ Recovery of heat-stressed cells	T^178^, S^180^	[68]
**Mrc1**	Radioactive	NS	Bacteria	Anti- p-S/T	Heat stress	Delay in DNA replication to avoid transcription-replication conflicts	T^169^, S^215^, S^229^	[69]
**Sic1**	Anti-p-Sic1 (T^163^)	Rapa	Bacteria	Anti- p-Sic1 (T^163^) Phos-tag	Rapa	Stabilization/G1-S arrest	T^163^	[70]
**Rcn2**	AS kinase	Cell wall stress (CR)	Bacteria Yeast	Phospho-peptide increase**^h^**	Pkc1 **^e^**	ND	S^152^, S^160^, S^255^	[50,54]
**Gga1**	AS kinase	Cell wall stress (CR)	Bacteria Yeast	Phospho-peptide Increase**^h^**	Pkc1 **^e^**	ND	ND	[50,54]
**Caf20**	AS kinase	Cell wall stress (CR)	Bacteria Yeast	Phospho-peptide increase**^h^**	Pkc1 **^e^**	ND	T^102^	[50,54]

ND (not determined), NS (not specified), CR (Congo red), CFW (calcofluor white), Rapa (rapamycin), Vanadate (sodium orthovanadate). **^a^**: Stimulus used for Slt2 activation. **^b^**: In vivo phosphorylation assay or alternative evidence (epistatic or physical interaction). **^c^**: Phosphorylation assay on a phosphatase dead version of Msg5 (Msg5^C319A^) [58]. **^d^**: Expression of a constitutively active allele of Bck1 (*BCK1*-20) [71]. **^e^**: Overexpression of a constitutively active allele of Pkc1 (PKC1 ^R398A, R405A, K406K^) [16]. **^f^**: Putative S/T-P sites. **^g^**: Phosphorylation assay on a protein fragment (Avo3 ^1−100^, Rlm1^329−445^, Med13 ^571−906^, Rpb1^1556−1718^, Sir3 ^1−439^). **^h^**: Slt2-dependence was not tested.

## Data Availability

The data presented in this study are available on request from the corresponding authors.

## Supplementary Materials

The following supporting information can be downloaded at: https://www.mdpi.com/article/10.3390/jof8040368/s1, Table S1: Peptides phosphorylated by Slt2 within the substrates listed in Table 1 and whose alignment has been performed in search for the consensus phosphorylation site shown in Figure 2.

## References

[1] Kock C., Dufrêne Y.F., Heinisch J.J. (2015). Up against the wall: Is yeast cell wall integrity ensured by mechanosensing in plasma membrane microdomains?. Appl. Environ. Microbiol..

[2] Levin D.E. (2011). Regulation of cell wall biogenesis in *Saccharomyces cerevisiae*: The cell wall integrity signaling pathway. Genetics.

[3] Chen R.E., Thorner J. (2007). Function and regulation in MAPK signaling pathways: Lessons learned from the yeast *Saccharomyces cerevisiae*. Biochim. Biophys. Acta.

[4] Schmelzle T., Helliwell S.B., Hall M.N. (2002). Yeast protein kinases and the RHO1 exchange factor TUS1 are novel components of the cell integrity pathway in yeast. Mol. Cell Biol..

[5] Heinisch J.J., Rodicio R. (2018). Protein kinase C in fungi-more than just cell wall integrity. FEMS Microbiol. Rev..

[6] Roelants F.M., Leskoske K.L., Martinez Marshall M.N., Locke M.N., Thorner J. (2017). The TORC2-Dependent Signaling Network in the Yeast *Saccharomyces cerevisiae*. Biomolecules.

[7] Inagaki M., Schmelzle T., Yamaguchi K., Irie K., Hall M.N., Matsumoto K. (1999). PDK1 homologs activate the Pkc1-mitogen-activated protein kinase pathway in yeast. Mol. Cell Biol..

[8] Martín H., Flandez M., Nombela C., Molina M. (2005). Protein phosphatases in MAPK signalling: We keep learning from yeast. Mol. Microbiol..

[9] Tatjer L., Sacristán-Reviriego A., Casado C., González A., Rodríguez-Porrata B., Palacios L., Canadell D., Serra-Cardona A., Martín H., Molina M. (2016). Wide-Ranging Effects of the Yeast Ptc1 Protein Phosphatase Acting Through the MAPK Kinase Mkk1. Genetics.

[10] González-Rubio G., Fernández-Acero T., Martín H., Molina M. (2019). Mitogen-Activated Protein Kinase Phosphatases (MKPs) in Fungal Signaling: Conservation, Function, and Regulation. Int. J. Mol. Sci..

[11] Molina M., Cid V.J., Martín H. (2010). Fine regulation of *Saccharomyces cerevisiae* MAPK pathways by post-translational modifications. Yeast.

[12] Audhya A., Emr S.D. (2002). Stt4 PI 4-kinase localizes to the plasma membrane and functions in the Pkc1-mediated MAP kinase cascade. Dev. Cell.

[13] Millson S.H., Truman A.W., King V., Prodromou C., Pearl L.H., Piper P.W. (2005). A two-hybrid screen of the yeast proteome for Hsp90 interactors uncovers a novel Hsp90 chaperone requirement in the activity of a stress-activated mitogen-activated protein kinase, Slt2p (Mpk1p). Eukaryot. Cell.

[14] Lee J., Liu L., Levin D.E. (2019). Stressing out or stressing in: Intracellular pathways for SAPK activation. Curr. Genet..

[15] Jiménez-Gutiérrez E., Alegría-Carrasco E., Sellers-Moya A., Molina M., Martín H. (2020). Not just the wall: The other ways to turn the yeast CWI pathway on. Int. Microbiol..

[16] Martín H., Rodríguez-Pachón J.M., Ruiz C., Nombela C., Molina M. (2000). Regulatory mechanisms for modulation of signaling through the cell integrity Slt2-mediated pathway in *Saccharomyces cerevisiae*. J. Biol. Chem..

[17] Liu L., Levin D.E. (2018). Intracellular mechanism by which genotoxic stress activates yeast SAPK Mpk1. Mol. Biol. Cell.

[18] González-Rubio G., Sellers-Moya Á., Martín H., Molina M. (2021). Differential Role of Threonine and Tyrosine Phosphorylation in the Activation and Activity of the Yeast MAPK Slt2. Int. J. Mol. Sci..

[19] Ahmadpour D., Maciaszczyk-Dziubinska E., Babazadeh R., Dahal S., Migocka M., Andersson M., Wysocki R., Tamás M.J., Hohmann S. (2016). The mitogen-activated protein kinase Slt2 modulates arsenite transport through the aquaglyceroporin Fps1. FEBS Lett..

[20] Pujol-Carrion N., Pavón-Vergés M., Arroyo J., de la Torre-Ruiz M.A. (2021). The MAPK Slt2/Mpk1 plays a role in iron homeostasis through direct regulation of the transcription factor Aft1. Biochim. Biophys. Acta Mol. Cell Res..

[21] Quilis I., Gomar-Alba M., Igual J.C. (2021). The CWI Pathway: A Versatile Toolbox to Arrest Cell-Cycle Progression. J. Fungi.

[22] Mao K., Klionsky D.J. (2011). MAPKs regulate mitophagy in *Saccharomyces cerevisiae*. Autophagy.

[23] Du Y., Walker L., Novick P., Ferro-Novick S. (2006). Ptc1p regulates cortical ER inheritance via Slt2p. Embo J..

[24] Li X., Du Y., Siegel S., Ferro-Novick S., Novick P. (2010). Activation of the mitogen-activated protein kinase, Slt2p, at bud tips blocks a late stage of endoplasmic reticulum inheritance in *Saccharomyces cerevisiae*. Mol. Biol. Cell.

[25] Guo S., Shen X., Yan G., Ma D., Bai X., Li S., Jiang Y. (2009). A MAP kinase dependent feedback mechanism controls Rho1 GTPase and actin distribution in yeast. PLoS ONE.

[26] García R., Sanz A.B., Rodríguez-Peña J.M., Nombela C., Arroyo J. (2016). Rlm1 mediates positive autoregulatory transcriptional feedback that is essential for Slt2-dependent gene expression. J. Cell Sci..

[27] Jiménez-Gutiérrez E., Alegría-Carrasco E., Alonso-Rodríguez E., Fernández-Acero T., Molina M., Martín H. (2020). Rewiring the yeast cell wall integrity (CWI) pathway through a synthetic positive feedback circuit unveils a novel role for the MAPKKK Ssk2 in CWI pathway activation. FEBS J..

[28] Soulard A., Cremonesi A., Moes S., Schütz F., Jenö P., Hall M.N. (2010). The rapamycin-sensitive phosphoproteome reveals that TOR controls protein kinase A toward some but not all substrates. Mol. Biol. Cell.

[29] Leskoske K.L., Roelants F.M., Emmerstorfer-Augustin A., Augustin C.M., Si E.P., Hill J.M., Thorner J. (2018). Phosphorylation by the stress-activated MAPK Slt2 down-regulates the yeast TOR complex 2. Genes Dev..

[30] Kim K.Y., Levin D.E. (2010). Transcriptional reporters for genes activated by cell wall stress through a non-catalytic mechanism involving Mpk1 and SBF. Yeast.

[31] Kim K.Y., Truman A.W., Levin D.E. (2008). Yeast Mpk1 mitogen-activated protein kinase activates transcription through Swi4/Swi6 by a noncatalytic mechanism that requires upstream signal. Mol. Cell Biol..

[32] Roskoski R. (2012). ERK1/2 MAP kinases: Structure, function, and regulation. Pharm. Res..

[33] Carlson S.M., Chouinard C.R., Labadorf A., Lam C.J., Schmelzle K., Fraenkel E., White F.M. (2011). Large-scale discovery of ERK2 substrates identifies ERK-mediated transcriptional regulation by ETV3. Sci. Signal..

[34] Mok J., Kim P.M., Lam H.Y., Piccirillo S., Zhou X., Jeschke G.R., Sheridan D.L., Parker S.A., Desai V., Jwa M. (2010). Deciphering protein kinase specificity through large-scale analysis of yeast phosphorylation site motifs. Sci. Signal..

[35] Bradley D., Beltrao P. (2019). Evolution of protein kinase substrate recognition at the active site. PLoS Biol..

[36] Tanoue T., Nishida E. (2002). Docking interactions in the mitogen-activated protein kinase cascades. Pharmacol. Ther..

[37] Garai Á., Zeke A., Gógl G., Törő I., Fördős F., Blankenburg H., Bárkai T., Varga J., Alexa A., Emig D. (2012). Specificity of linear motifs that bind to a common mitogen-activated protein kinase docking groove. Sci. Signal..

[38] Tanoue T., Adachi M., Moriguchi T., Nishida E. (2000). A conserved docking motif in MAP kinases common to substrates, activators and regulators. Nat. Cell Biol..

[39] Chang C.I., Xu B.E., Akella R., Cobb M.H., Goldsmith E.J. (2002). Crystal structures of MAP kinase p38 complexed to the docking sites on its nuclear substrate MEF2A and activator MKK3b. Mol. Cell.

[40] Zeke A., Bastys T., Alexa A., Garai Á., Mészáros B., Kirsch K., Dosztányi Z., Kalinina O.V., Reményi A. (2015). Systematic discovery of linear binding motifs targeting an ancient protein interaction surface on MAP kinases. Mol. Syst. Biol..

[41] Akella R., Moon T.M., Goldsmith E.J. (2008). Unique MAP Kinase binding sites. Biochim. Biophys. Acta.

[42] González-Rubio G., Sellers-Moya Á., Martín H., Molina M. (2021). A walk-through MAPK structure and functionality with the 30-year-old yeast MAPK Slt2. Int. Microbiol..

[43] Watanabe Y., Irie K., Matsumoto K. (1995). Yeast RLM1 encodes a serum response factor-like protein that may function downstream of the Mpk1 (Slt2) mitogen-activated protein kinase pathway. Mol. Cell Biol..

[44] Watanabe Y., Takaesu G., Hagiwara M., Irie K., Matsumoto K. (1997). Characterization of a serum response factor-like protein in *Saccharomyces cerevisiae*, Rlm1, which has transcriptional activity regulated by the Mpk1 (Slt2) mitogen-activated protein kinase pathway. Mol. Cell Biol..

[45] Jia Y., Quinn C.M., Kwak S., Talanian R.V. (2008). Current in vitro kinase assay technologies: The quest for a universal format. Curr. Drug Discov. Technol..

[46] Hastie C.J., McLauchlan H.J., Cohen P. (2006). Assay of protein kinases using radiolabeled ATP: A protocol. Nat. Protoc..

[47] Mok J., Im H., Snyder M. (2009). Global identification of protein kinase substrates by protein microarray analysis. Nat. Protoc..

[48] Shah K., Shokat K.M. (2003). A chemical genetic approach for the identification of direct substrates of protein kinases. Methods Mol. Biol..

[49] Hertz N.T., Wang B.T., Allen J.J., Zhang C., Dar A.C., Burlingame A.L., Shokat K.M. (2010). Chemical genetic approach for kinase-substrate mapping by covalent capture of thiophosphopeptides and analysis by mass spectrometry. Curr. Protoc. Chem. Biol..

[50] Alonso-Rodríguez E., Fernández-Pinar P., Sacristán-Reviriego A., Molina M., Martín H. (2016). An Analog-sensitive Version of the Protein Kinase Slt2 Allows Identification of Novel Targets of the Yeast Cell Wall Integrity Pathway. J. Biol. Chem..

[51] Koch A., Hauf S. (2010). Strategies for the identification of kinase substrates using analog-sensitive kinases. Eur J. Cell Biol..

[52] Sugiyama Y., Uezato Y. (2022). Analysis of protein kinases by Phos-tag SDS-PAGE. J. Proteom..

[53] von Stechow L., Francavilla C., Olsen J.V. (2015). Recent findings and technological advances in phosphoproteomics for cells and tissues. Expert Rev. Proteom..

[54] Mascaraque V., Hernaez M.L., Jimenez-Sanchez M., Hansen R., Gil C., Martin H., Cid V.J., Molina M. (2013). Phosphoproteomic analysis of protein kinase C signaling in *Saccharomyces cerevisiae* reveals Slt2 mitogen-activated protein kinase (MAPK)-dependent phosphorylation of eisosome core components. Mol. Cell Proteom..

[55] Knight J.D., Tian R., Lee R.E., Wang F., Beauvais A., Zou H., Megeney L.A., Gingras A.C., Pawson T., Figeys D. (2012). A novel whole-cell lysate kinase assay identifies substrates of the p38 MAPK in differentiating myoblasts. Skelet Muscle.

[56] van Drogen F., Peter M. (2002). Spa2p functions as a scaffold-like protein to recruit the Mpk1p MAP kinase module to sites of polarized growth. Curr. Biol..

[57] Jiménez-Sánchez M., Cid V.J., Molina M. (2007). Retrophosphorylation of Mkk1 and Mkk2 MAPKKs by the Slt2 MAPK in the yeast cell integrity pathway. J. Biol. Chem..

[58] Flández M., Cosano I.C., Nombela C., Martín H., Molina M. (2004). Reciprocal regulation between Slt2 MAPK and isoforms of Msg5 dual-specificity protein phosphatase modulates the yeast cell integrity pathway. J. Biol. Chem..

[59] Marín M.J., Flández M., Bermejo C., Arroyo J., Martín H., Molina M. (2009). Different modulation of the outputs of yeast MAPK-mediated pathways by distinct stimuli and isoforms of the dual-specificity phosphatase Msg5. Mol. Genet. Genom..

[60] Madden K., Sheu Y.J., Baetz K., Andrews B., Snyder M. (1997). SBF cell cycle regulator as a target of the yeast PKC-MAP kinase pathway. Science.

[61] Kim K.Y., Truman A.W., Caesar S., Schlenstedt G., Levin D.E. (2010). Yeast Mpk1 cell wall integrity mitogen-activated protein kinase regulates nucleocytoplasmic shuttling of the Swi6 transcriptional regulator. Mol. Biol. Cell.

[62] Jin C., Strich R., Cooper K.F. (2014). Slt2p phosphorylation induces cyclin C nuclear-to-cytoplasmic translocation in response to oxidative stress. Mol. Biol. Cell.

[63] Krasley E., Cooper K.F., Mallory M.J., Dunbrack R., Strich R. (2006). Regulation of the oxidative stress response through Slt2p-dependent destruction of cyclin C in *Saccharomyces cerevisiae*. Genetics.

[64] Stieg D.C., Willis S.D., Ganesan V., Ong K.L., Scuorzo J., Song M., Grose J., Strich R., Cooper K.F. (2018). A complex molecular switch directs stress-induced cyclin C nuclear release through SCF(Grr1)-mediated degradation of Med13. Mol. Biol. Cell.

[65] Yurko N., Liu X., Yamazaki T., Hoque M., Tian B., Manley J.L. (2017). MPK1/SLT2 Links Multiple Stress Responses with Gene Expression in Budding Yeast by Phosphorylating Tyr1 of the RNAP II CTD. Mol. Cell.

[66] Ai W., Bertram P.G., Tsang C.K., Chan T.F., Zheng X.F. (2002). Regulation of subtelomeric silencing during stress response. Mol. Cell.

[67] Ray A., Hector R.E., Roy N., Song J.H., Berkner K.L., Runge K.W. (2003). Sir3p phosphorylation by the Slt2p pathway effects redistribution of silencing function and shortened lifespan. Nat. Genet..

[68] Carmody S.R., Tran E.J., Apponi L.H., Corbett A.H., Wente S.R. (2010). The mitogen-activated protein kinase Slt2 regulates nuclear retention of non-heat shock mRNAs during heat shock-induced stress. Mol. Cell Biol..

[69] Duch A., Canal B., Barroso S.I., García-Rubio M., Seisenbacher G., Aguilera A., de Nadal E., Posas F. (2018). Multiple signaling kinases target Mrc1 to prevent genomic instability triggered by transcription-replication conflicts. Nat. Commun..

[70] Moreno-Torres M., Jaquenoud M., De Virgilio C. (2015). TORC1 controls G1-S cell cycle transition in yeast via Mpk1 and the greatwall kinase pathway. Nat. Commun..

[71] Lee K.S., Levin D.E. (1992). Dominant mutations in a gene encoding a putative protein kinase (BCK1) bypass the requirement for a *Saccharomyces cerevisiae* protein kinase C homolog. Mol. Cell Biol..

[72] Ozaki K., Tanaka K., Imamura H., Hihara T., Kameyama T., Nonaka H., Hirano H., Matsuura Y., Takai Y. (1996). Rom1p and Rom2p are GDP/GTP exchange proteins (GEPs) for the Rho1p small GTP binding protein in *Saccharomyces cerevisiae*. EMBO J..

[73] Manning B.D., Padmanabha R., Snyder M. (1997). The Rho-GEF Rom2p localizes to sites of polarized cell growth and participates in cytoskeletal functions in *Saccharomyces cerevisiae*. Mol. Biol. Cell.

[74] Kobayashi T., Takematsu H., Yamaji T., Hiramoto S., Kozutsumi Y. (2005). Disturbance of sphingolipid biosynthesis abrogates the signaling of Mss4, phosphatidylinositol-4-phosphate 5-kinase, in yeast. J. Biol. Chem..

[75] Yamochi W., Tanaka K., Nonaka H., Maeda A., Musha T., Takai Y. (1994). Growth site localization of Rho1 small GTP-binding protein and its involvement in bud formation in *Saccharomyces cerevisiae*. J. Cell Biol..

[76] Holt L.J., Tuch B.B., Villén J., Johnson A.D., Gygi S.P., Morgan D.O. (2009). Global analysis of Cdk1 substrate phosphorylation sites provides insights into evolution. Science.

[77] Saccharomyces Genome Database. https://www.yeastgenome.org/.

[78] Soler M., Plovins A., Martín H., Molina M., Nombela C. (1995). Characterization of domains in the yeast MAP kinase Slt2 (Mpk1) required for functional activity and in vivo interaction with protein kinases Mkk1 and Mkk2. Mol. Microbiol..

[79] Lee K.S., Irie K., Gotoh Y., Watanabe Y., Araki H., Nishida E., Matsumoto K., Levin D.E. (1993). A yeast mitogen-activated protein kinase homolog (Mpk1p) mediates signalling by protein kinase C. Mol. Cell Biol..

[80] Irie K., Takase M., Lee K.S., Levin D.E., Araki H., Matsumoto K., Oshima Y. (1993). MKK1 and MKK2, which encode *Saccharomyces cerevisiae* mitogen-activated protein kinase-kinase homologs, function in the pathway mediated by protein kinase C. Mol. Cell Biol..

[81] Errede B., Gartner A., Zhou Z., Nasmyth K., Ammerer G. (1993). MAP kinase-related FUS3 from S. cerevisiae is activated by STE7 in vitro. Nature.

[82] Zhou Z., Gartner A., Cade R., Ammerer G., Errede B. (1993). Pheromone-induced signal transduction in *Saccharomyces cerevisiae* requires the sequential function of three protein kinases. Mol. Cell Biol..

[83] Buscà R., Pouysségur J., Lenormand P. (2016). ERK1 and ERK2 Map Kinases: Specific Roles or Functional Redundancy?. Front. Cell Dev. Biol..

[84] Katagiri C., Masuda K., Urano T., Yamashita K., Araki Y., Kikuchi K., Shima H. (2005). Phosphorylation of Ser-446 determines stability of MKP-7. J. Biol. Chem..

[85] Brondello J.M., Pouysségur J., McKenzie F.R. (1999). Reduced MAP kinase phosphatase-1 degradation after p42/p44MAPK-dependent phosphorylation. Science.

[86] Sohaskey M.L., Ferrell J.E. (2002). Activation of p42 mitogen-activated protein kinase (MAPK), but not c-Jun NH(2)-terminal kinase, induces phosphorylation and stabilization of MAPK phosphatase XCL100 in Xenopus oocytes. Mol. Biol. Cell.

[87] Portela P., Rossi S. (2020). cAMP-PKA signal transduction specificity in *Saccharomyces cerevisiae*. Curr. Genet..

[88] Santangelo G.M. (2006). Glucose signaling in *Saccharomyces cerevisiae*. Microbiol. Mol. Biol. Rev..

[89] Johnson K.E., Cameron S., Toda T., Wigler M., Zoller M.J. (1987). Expression in Escherichia coli of BCY1, the regulatory subunit of cyclic AMP-dependent protein kinase from Saccharomyces cerevisiae. Purification and characterization. J. Biol. Chem..

[90] Kuret J., Johnson K.E., Nicolette C., Zoller M.J. (1988). Mutagenesis of the regulatory subunit of yeast cAMP-dependent protein kinase. Isolation of site-directed mutants with altered binding affinity for catalytic subunit. J. Biol. Chem..

[91] Griffioen G., Branduardi P., Ballarini A., Anghileri P., Norbeck J., Baroni M.D., Ruis H. (2001). Nucleocytoplasmic distribution of budding yeast protein kinase A regulatory subunit Bcy1 requires Zds1 and is regulated by Yak1-dependent phosphorylation of its targeting domain. Mol. Cell Biol..

[92] Griffioen G., Swinnen S., Thevelein J.M. (2003). Feedback inhibition on cell wall integrity signaling by Zds1 involves Gsk3 phosphorylation of a cAMP-dependent protein kinase regulatory subunit. J. Biol. Chem..

[93] Searle J.S., Wood M.D., Kaur M., Tobin D.V., Sanchez Y. (2011). Proteins in the nutrient-sensing and DNA damage checkpoint pathways cooperate to restrain mitotic progression following DNA damage. PLoS Genet..

[94] Locke M.N., Thorner J. (2019). Regulation of TORC2 function and localization by Rab5 GTPases in *Saccharomyces cerevisiae*. Cell Cycle.

[95] Turjanski A.G., Vaqué J.P., Gutkind J.S. (2007). MAP kinases and the control of nuclear events. Oncogene.

[96] Sanz A.B., García R., Pavón-Vergés M., Rodríguez-Peña J.M., Arroyo J. (2022). Control of Gene Expression via the Yeast CWI Pathway. Int. J. Mol. Sci..

[97] García R., Bermejo C., Grau C., Pérez R., Rodríguez-Peña J.M., Francois J., Nombela C., Arroyo J. (2004). The global transcriptional response to transient cell wall damage in *Saccharomyces cerevisiae* and its regulation by the cell integrity signaling pathway. J. Biol. Chem..

[98] Jung U.S., Sobering A.K., Romeo M.J., Levin D.E. (2002). Regulation of the yeast Rlm1 transcription factor by the Mpk1 cell wall integrity MAP kinase. Mol. Microbiol..

[99] Sanz A.B., Garcia R., Rodriguez-Pena J.M., Nombela C., Arroyo J. (2018). Slt2 MAPK association with chromatin is required for transcriptional activation of Rlm1 dependent genes upon cell wall stress. Biochim. Biophys. Acta Gene Regul. Mech..

[100] Haase S.B., Wittenberg C. (2014). Topology and control of the cell-cycle-regulated transcriptional circuitry. Genetics.

[101] Baetz K., Andrews B. (1999). Regulation of cell cycle transcription factor Swi4 through auto-inhibition of DNA binding. Mol. Cell Biol..

[102] Igual J.C., Johnson A.L., Johnston L.H. (1996). Coordinated regulation of gene expression by the cell cycle transcription factor Swi4 and the protein kinase C MAP kinase pathway for yeast cell integrity. EMBO J..

[103] Truman A.W., Kim K.Y., Levin D.E. (2009). Mechanism of Mpk1 mitogen-activated protein kinase binding to the Swi4 transcription factor and its regulation by a novel caffeine-induced phosphorylation. Mol. Cell Biol..

[104] Schier A.C., Taatjes D.J. (2020). Structure and mechanism of the RNA polymerase II transcription machinery. Genes Dev..

[105] Kornberg R.D. (2005). Mediator and the mechanism of transcriptional activation. Trends BioChem. Sci..

[106] Harper T.M., Taatjes D.J. (2018). The complex structure and function of Mediator. J. Biol. Chem..

[107] Ježek J., Smethurst D.G.J., Stieg D.C., Kiss Z.A.C., Hanley S.E., Ganesan V., Chang K.T., Cooper K.F., Strich R. (2019). Cyclin C: The Story of a Non-Cycling Cyclin. Biology.

[108] Cooper K.F., Mallory M.J., Smith J.B., Strich R. (1997). Stress and developmental regulation of the yeast C-type cyclin Ume3p (Srb11p/Ssn8p). EMBO J..

[109] Cooper K.F., Mallory M.J., Strich R. (1999). Oxidative stress-induced destruction of the yeast C-type cyclin Ume3p requires phosphatidylinositol-specific phospholipase C and the 26S proteasome. Mol. Cell Biol..

[110] Cooper K.F., Khakhina S., Kim S.K., Strich R. (2014). Stress-induced nuclear-to-cytoplasmic translocation of cyclin C promotes mitochondrial fission in yeast. Dev. Cell.

[111] Cooper K.F., Scarnati M.S., Krasley E., Mallory M.J., Jin C., Law M.J., Strich R. (2012). Oxidative-stress-induced nuclear to cytoplasmic relocalization is required for Not4-dependent cyclin C destruction. J. Cell Sci..

[112] Levin-Salomon V., Maayan I., Avrahami-Moyal L., Marbach I., Livnah O., Engelberg D. (2009). When expressed in yeast, mammalian mitogen-activated protein kinases lose proper regulation and become spontaneously phosphorylated. BioChem. J..

[113] Valencia A.M., Kadoch C. (2019). Chromatin regulatory mechanisms and therapeutic opportunities in cancer. Nat. Cell Biol..

[114] Gartenberg M.R., Smith J.S. (2016). The Nuts and Bolts of Transcriptionally Silent Chromatin in *Saccharomyces cerevisiae*. Genetics.

[115] Sauty S.M., Shaban K., Yankulov K. (2021). Gene repression in S. cerevisiae-looking beyond Sir-dependent gene silencing. Curr. Genet..

[116] Stone E.M., Pillus L. (1996). Activation of an MAP kinase cascade leads to Sir3p hyperphosphorylation and strengthens transcriptional silencing. J. Cell Biol..

[117] Viswanathan M., Muthukumar G., Cong Y.S., Lenard J. (1994). Seripauperins of *Saccharomyces cerevisiae*: A new multigene family encoding serine-poor relatives of serine-rich proteins. Gene.

[118] Kothiwal D., Laloraya S. (2019). A SIR-independent role for cohesin in subtelomeric silencing and organization. Proc. Natl. Acad. Sci. USA.

[119] Blasl A.T., Schulze S., Qin C., Graf L.G., Vogt R., Lammers M. (2022). Post-translational lysine ac(et)ylation in health, ageing and disease. Biol. Chem..

[120] Strahl-Bolsinger S., Hecht A., Luo K., Grunstein M. (1997). SIR2 and SIR4 interactions differ in core and extended telomeric heterochromatin in yeast. Genes Dev..

[121] Lin S.J., Kaeberlein M., Andalis A.A., Sturtz L.A., Defossez P.A., Culotta V.C., Fink G.R., Guarente L. (2002). Calorie restriction extends *Saccharomyces cerevisiae* lifespan by increasing respiration. Nature.

[122] Lu Y.Y., Krebber H. (2021). Nuclear mRNA Quality Control and Cytoplasmic NMD Are Linked by the Guard Proteins Gbp2 and Hrb1. Int. J. Mol. Sci..

[123] Saavedra C., Tung K.S., Amberg D.C., Hopper A.K., Cole C.N. (1996). Regulation of mRNA export in response to stress in *Saccharomyces cerevisiae*. Genes Dev..

[124] Zarnack K., Balasubramanian S., Gantier M.P., Kunetsky V., Kracht M., Schmitz M.L., Sträßer K. (2020). Dynamic mRNP Remodeling in Response to Internal and External Stimuli. Biomolecules.

[125] Alpert T., Straube K., Carrillo Oesterreich F., Herzel L., Neugebauer K.M. (2020). Widespread Transcriptional Readthrough Caused by Nab2 Depletion Leads to Chimeric Transcripts with Retained Introns. Cell Rep..

[126] Zinzalla V., Graziola M., Mastriani A., Vanoni M., Alberghina L. (2007). Rapamycin-mediated G1 arrest involves regulation of the Cdk inhibitor Sic1 in *Saccharomyces cerevisiae*. Mol. Microbiol..

[127] Escoté X., Zapater M., Clotet J., Posas F. (2004). Hog1 mediates cell-cycle arrest in G1 phase by the dual targeting of Sic1. Nat. Cell Biol..

[128] Moreno-Torres M., Jaquenoud M., Péli-Gulli M.P., Nicastro R., De Virgilio C. (2017). TORC1 coordinates the conversion of Sic1 from a target to an inhibitor of cyclin-CDK-Cks1. Cell Discov..

[129] Kono K., Al-Zain A., Schroeder L., Nakanishi M., Ikui A.E. (2016). Plasma membrane/cell wall perturbation activates a novel cell cycle checkpoint during G1 in *Saccharomyces cerevisiae*. Proc. Natl. Acad. Sci. USA.

[130] Yeeles J.T.P., Janska A., Early A., Diffley J.F.X. (2017). How the Eukaryotic Replisome Achieves Rapid and Efficient DNA Replication. Mol. Cell.

[131] Katou Y., Kanoh Y., Bando M., Noguchi H., Tanaka H., Ashikari T., Sugimoto K., Shirahige K. (2003). S-phase checkpoint proteins Tof1 and Mrc1 form a stable replication-pausing complex. Nature.

[132] Uzunova S.D., Zarkov A.S., Ivanova A.M., Stoynov S.S., Nedelcheva-Veleva M.N. (2014). The subunits of the S-phase checkpoint complex Mrc1/Tof1/Csm3: Dynamics and interdependence. Cell Div..

[133] Voordeckers K., Colding C., Grasso L., Pardo B., Hoes L., Kominek J., Gielens K., Dekoster K., Gordon J., Van der Zande E. (2020). Ethanol exposure increases mutation rate through error-prone polymerases. Nat. Commun..

[134] Duch A., Felipe-Abrio I., Barroso S., Yaakov G., García-Rubio M., Aguilera A., de Nadal E., Posas F. (2013). Coordinated control of replication and transcription by a SAPK protects genomic integrity. Nature.

[135] Castelli L.M., Talavera D., Kershaw C.J., Mohammad-Qureshi S.S., Costello J.L., Rowe W., Sims P.F., Grant C.M., Hubbard S.J., Ashe M.P. (2015). The 4E-BP Caf20p Mediates Both eIF4E-Dependent and Independent Repression of Translation. PLoS Genet..

[136] Mehta S., Li H., Hogan P.G., Cunningham K.W. (2009). Domain architecture of the regulators of calcineurin (RCANs) and identification of a divergent RCAN in yeast. Mol. Cell Biol..

[137] MacGilvray M.E., Shishkova E., Place M., Wagner E.R., Coon J.J., Gasch A.P. (2020). Phosphoproteome Response to Dithiothreitol Reveals Unique Versus Shared Features of *Saccharomyces cerevisiae* Stress Responses. J. Proteome Res..

[138] Bonilla M., Nastase K.K., Cunningham K.W. (2002). Essential role of calcineurin in response to endoplasmic reticulum stress. EMBO J..

[139] Chen Y., Feldman D.E., Deng C., Brown J.A., De Giacomo A.F., Gaw A.F., Shi G., Le Q.T., Brown J.M., Koong A.C. (2005). Identification of mitogen-activated protein kinase signaling pathways that confer resistance to endoplasmic reticulum stress in *Saccharomyces cerevisiae*. Mol. Cancer Res..

[140] Crooks G.E., Hon G., Chandonia J.M., Brenner S.E. (2004). WebLogo: A sequence logo generator. Genome Res..

[141] Ptacek J., Devgan G., Michaud G., Zhu H., Zhu X., Fasolo J., Guo H., Jona G., Breitkreutz A., Sopko R. (2005). Global analysis of protein phosphorylation in yeast. Nature.

[142] Shimoji K., Jakovljevic J., Tsuchihashi K., Umeki Y., Wan K., Kawasaki S., Talkish J., Woolford J.L., Mizuta K. (2012). Ebp2 and Brx1 function cooperatively in 60S ribosomal subunit assembly in *Saccharomyces cerevisiae*. Nucleic Acids Res..

[143] Xu H., Fang T., Yan H., Jiang L. (2019). The protein kinase Cmk2 negatively regulates the calcium/calcineurin signalling pathway and expression of calcium pump genes PMR1 and PMC1 in budding yeast. Cell Commun. Signal..

[144] Lanze C.E., Gandra R.M., Foderaro J.E., Swenson K.A., Douglas L.M., Konopka J.B. (2020). Plasma Membrane MCC/Eisosome Domains Promote Stress Resistance in Fungi. Microbiol. Mol. Biol. Rev..

[145] Luo G., Gruhler A., Liu Y., Jensen O.N., Dickson R.C. (2008). The sphingolipid long-chain base-Pkh1/2-Ypk1/2 signaling pathway regulates eisosome assembly and turnover. J. Biol. Chem..

[146] Walther T.C., Aguilar P.S., Fröhlich F., Chu F., Moreira K., Burlingame A.L., Walter P. (2007). Pkh-kinases control eisosome assembly and organization. EMBO J..

[147] Pearce L.R., Komander D., Alessi D.R. (2010). The nuts and bolts of AGC protein kinases. Nat. Rev. Mol. Cell Biol..

[148] Foderaro J.E., Douglas L.M., Konopka J.B. (2017). MCC/Eisosomes Regulate Cell Wall Synthesis and Stress Responses in Fungi. J. Fungi.

[149] Kozubowski L., Panek H., Rosenthal A., Bloecher A., DeMarini D.J., Tatchell K. (2003). A Bni4-Glc7 phosphatase complex that recruits chitin synthase to the site of bud emergence. Mol. Biol. Cell.

[150] Pérez J., Arcones I., Gómez A., Casquero V., Roncero C. (2016). Phosphorylation of Bni4 by MAP kinases contributes to septum assembly during yeast cytokinesis. FEMS Yeast Res..

[151] Dokládal L., Stumpe M., Hu Z., Jaquenoud M., Dengjel J., De Virgilio C. (2021). Phosphoproteomic responses of TORC1 target kinases reveal discrete and convergent mechanisms that orchestrate the quiescence program in yeast. Cell Rep..

[152] Garrett-Engele P., Moilanen B., Cyert M.S. (1995). Calcineurin, the Ca2+/calmodulin-dependent protein phosphatase, is essential in yeast mutants with cell integrity defects and in mutants that lack a functional vacuolar H(+)-ATPase. Mol. Cell Biol..

[153] Kim K.Y., Cosano I.C., Levin D.E., Molina M., Martin H. (2007). Dissecting the transcriptional activation function of the cell wall integrity MAP kinase. Yeast.

[154] Casar B., Pinto A., Crespo P. (2008). Essential role of ERK dimers in the activation of cytoplasmic but not nuclear substrates by ERK-scaffold complexes. Mol. Cell.

