# Supplementary material for: Substrates of the MAPK Slt2: Shaping Yeast Cell Integrity
Source: J Fungi (Basel). 2022 Apr 4;8(4):368. doi: 10.3390/jof8040368 (PMC9031059; doi:10.3390/jof8040368)
Supplement: Supplementary file 1 [file jof-08-00368-s001.zip › jof-1661885-supplementary.pdf]

Table S1:

Peptides phosphorylated by Slt2 within the substrates listed in Table1 and whose alignment has been performed in search for the consensus phosphorylation site shown in Figure 2. The name of the substrate and the precise phosphosite are indicated. The sequence of the peptide contains the phosphorylated S/T residue (underlined) and 10 amino acids upstream/downstream this position. Rpb1 phosphorylated peptides are not included in this list.

Substrate	Phosphosite	Phosphorylated peptide
**Mkk2**	**S50**	NRSPYSSVNESPYSNNSTSAT
**Rlm1**	**S234**	NLNQPSSSSSSPSTMDFPKLP
**Rlm1**	**S261**	FNGRPPPISISPNKFSKPFTN
**Rlm1**	**T276**	SKPFTNASSRTPKQEHKINNS
**Rlm1**	**S299**	NNNDNSNYTQSPSNSLEDSIQ
**Rlm1**	**S374**	ENKTSRSSKISPLSASASGPL
**Rlm1**	**S427**	NHPYPFGSGSSPLFSATQPYI
**Rlm1**	**T439**	LFSATQPYIATPLQPSNIPGG
**Rlm1**	**S518**	SKFVHDLMSNSPNVSSISMFP
**Rlm1**	**T646**	ATAYNGNTGLTPYINTAQTPL
**Rlm1**	**T654**	GLTPYINTAQTPLGTKFFNFS
**Bcy1**	**T129**	QEEQQHTREKTSTPPLPMHFN
**Avo2**	**T157**	DLWVMDTNGDTPLHVCLEYGS
**Avo2**	**T232**	GAGKKPSSFRTPILNAKATFE
**Avo2**	**S246**	NAKATFEDGPSPVLSMNSPYS
**Avo2**	**S253**	DGPSPVLSMNSPYSLYSNNSP
**Avo2**	**S262**	NSPYSLYSNNSPLPVLPRRIS
**Avo2**	**T323**	RLRVNSINVKTPVGVSPKKEL
**Avo2**	**T328**	SINVKTPVGVSPKKELVSESV
**Avo2**	**T343**	LVSESVRHSATPTSPHNNIAL
**Avo2**	**S346**	ESVRHSATPTSPHNNIALINR
**Avo3**	**S12**	MSIPHSAKQSSPLSSRRRSVT
**Avo3**	**T25**	SSRRRSVTNTTPLLTPRHSRD
**Avo3**	**T29**	RSVTNTTPLLTPRHSRDNSST
**Avo3**	**S51**	ISSAKNITSSSPSTITNESSK
**Avo3**	**S85**	STKRLENSAPSPTSPLMARRT
**Avo3**	**S88**	RLENSAPSPTSPLMARRTRST
**Mrc1**	**T169**	LVNETSQALKTPLTTGRPGAT
**Mrc1**	**S215**	TTSSNHSNALSPKIPIIPTEL
**Mrc1**	**S229**	PIIPTELIGTSPLFQSIQNRG
**Sic1**	**T163**	RKKIIKDVPGTPSDKVITFEL
**Swi6**	**S238**	SPLKIMKALPSPVVNDNEQKM
**Ssn8 (cyclin C)**	**S266**	EAIRDPKNSSSPVQIAFNRFM
**Med13**	**T835**	PLASIPDIFITPTPVVTISEK
**Med13**	**T837**	ASIPDIFITPTPVVTISEKEQ
**Sir3**	**S275**	RGTSTTHGSISPQEESVSPNI
**Sir3**	**S282**	GSISPQEESVSPNISSASPSA
**Sir3**	**S289**	ESVSPNISSASPSALTSPTDS
**Sir3**	**S295**	ISSASPSALTSPTDSSKILQK
**Nab2**	**T178**	QNAMQTDAPATPSPISAFSGV
**Nab2**	**S180**	AMQTDAPATPSPISAFSGVVN
**Rcn2**	**S152**	SSLNKGGSSLSPDKSSLESPT
**Rcn2**	**S160**	SLSPDKSSLESPTMLKLSTDS
**Caf20**	**T102**	GEDDEEETETTPTSTVPVATI
